# Host and microbiome jointly contribute to environmental adaptation

**DOI:** 10.1038/s41396-023-01507-9

**Published:** 2023-09-06

**Authors:** Carola Petersen, Inga K. Hamerich, Karen L. Adair, Hanne Griem-Krey, Montserrat Torres Oliva, Marc P. Hoeppner, Brendan J. M. Bohannan, Hinrich Schulenburg

**Affiliations:** 1 Department of Evolutionary Ecology and Genetics, Kiel University, Kiel, Germany; 2 Institute of Ecology and Evolution, University of Oregon, Eugene, OR USA; 3 Institute of Clinical Molecular Biology, Kiel University, Kiel, Germany; 4 Max-Planck Institute for Evolutionary Biology, Ploen, Germany

**Keywords:** Evolution, Microbial ecology, Microbiome

## Abstract

Most animals and plants have associated microorganisms, collectively referred to as their microbiomes, which can provide essential functions. Given their importance, host-associated microbiomes have the potential to contribute substantially to adaptation of the host-microbiome assemblage (the “metaorganism”). Microbiomes may be especially important for rapid adaptation to novel environments because microbiomes can change more rapidly than host genomes. However, it is not well understood how hosts and microbiomes jointly contribute to metaorganism adaptation. We developed a model system with which to disentangle the contributions of hosts and microbiomes to metaorganism adaptation. We established replicate mesocosms containing the nematode *Caenorhabditis elegans* co-cultured with microorganisms in a novel complex environment (laboratory compost). After approximately 30 nematode generations (100 days), we harvested worm populations and associated microbiomes, and subjected them to a common garden experiment designed to unravel the impacts of microbiome composition and host genetics on metaorganism adaptation. We observed that adaptation took different trajectories in different mesocosm lines, with some increasing in fitness and others decreasing, and that interactions between host and microbiome played an important role in these contrasting evolutionary paths. We chose two exemplary mesocosms (one with a fitness increase and one with a decrease) for detailed study. For each example, we identified specific changes in both microbiome composition (for both bacteria and fungi) and nematode gene expression associated with each change in fitness. Our study provides experimental evidence that adaptation to a novel environment can be jointly influenced by host and microbiome.

## Introduction

The microorganisms associated with most animals and plants are collectively known as their microbiomes. These microorganisms can provide important biological functions including digestion of otherwise indigestible materials [1], production of essential nutrients [2], increased resistance to pathogens [2–4], and stimulation of development (including maturation of the immune system) [5], among many others. Because of the potential impact of microbiomes on crucial physiological functions, it has been suggested that multicellular organisms are best conceptualized as “metaorganisms” or “holobionts” – multispecies assemblages with collective properties such as fitness [6, 7].

Given the importance of microbiomes to metaorganism function, they could play an important role in evolutionary adaptation, especially in response to environmental change. Microbiomes may be especially important in mediating acclimation and adaptation to environmental change because microbiomes can rapidly respond to environmental challenges, both through changes in microbiome composition and through genetic and phenotypically plastic changes in individual microbial lineages. Hosts may respond more slowly than their microbiomes to a changing environment because hosts often have longer generation times and smaller population sizes than their associated microorganisms [8].

Microbiome-mediated acclimation to environmental change has been documented in multiple metaorganisms, most frequently in response to increasing temperatures [9]. For example, some corals have been found to harbor heat-tolerant microbes, which can help the coral survive in warmer waters, as reported for *Acropora hyacinthus* [10]. Similarly, long-term exposure of the sea anemone *Nematostella vectensis* to increased temperatures led to both higher heat tolerance and microbiome changes; subsequent transplant experiments demonstrated that the higher heat tolerance was a consequence of changes in microbiome composition [11].

Even if more slowly, genetic changes in the host population (i.e., host evolution) could still improve performance of the metaorganism in the new environment. To date, a joint assessment of the contribution of either microbiome acclimation and/or host evolution has only rarely been attempted. One of the few examples is the study of pathogen stress in the nematode host *Caenorhabditis elegans. C. elegans*, together with a single symbiont *Enterococcus faecalis*, was exposed to pathogen stress over 14 host generations under controlled laboratory conditions. The symbiont was observed to evolve an increased protective effect and simultaneously the host evolved an increased ability to accommodate the protective symbiont [12–14]. A more recent example adapted the parasitoid wasp *Nasonia vitripennis* with its diverse microbiome over 85 generations to the herbicide atrazine, demonstrating that both changes in the microbiome, as well as genetic adaptations in the host, increased atrazine resistance in an interdependent manner, consistent with a co-adapted host-microbiome association [15]. In this example, it is as yet unclear whether the genetic changes in the host favored colonization with the beneficial microbes and/or directly mediated resistance [15]. Overall, the microbiome can play a central role in metaorganism acclimation to novel environmental conditions, yet to date the importance of host genetic adaptation in this context is poorly understood.

The aim of our study is to establish a novel experimental metaorganism system for studying the causes and consequences of microbiome-mediated acclimation and to specifically explore the contribution of both host and microbiome to improved performance in a novel environment. We developed and implemented a mesocosm experimental approach with the nematode *C. elegans* and its microbiome as a model. This nematode is common in temperate regions across the world, where it proliferates in rotting plant matter, especially rotting fruits or compost [16], where it associates with a species-rich gut microbiome, consisting of Proteobacteria such as Enterobacteriaceae and *Pseudomonas*, *Stenotrophomonas*, *Ochrobactrum*, and *Sphingomonas* bacteria, as well as certain yeast species [17, 18]. We maintained a genetically diverse *C. elegans* laboratory population in an experimental compost environment similar in many respects to the nematode’s natural habitat [16, 19]. After 100 days (approx. 30 host generations), we harvested and separated worm populations and associated microbiomes, and subjected them to two sets of common garden experiments designed to unravel the impacts of microbiome composition and host genetics on metaorganism adaptation. The first common garden experiment revealed that adaptation took different trajectories in different mesocosm lines, with some increasing in fitness and others decreasing. The second common garden experiment focused on two of the mesocosm lines and demonstrated that interactions between the host and microbiome played an important role in these contrasting evolutionary paths. For these two mesocosm lines, we further assessed the underlying changes in microbiome composition and host gene expression.

## Materials and Methods

### Nematode and bacterial strains

The mesocosm experiment was initiated with the experimental and genetically diverse, androdioecious *C. elegans* population A_0_ derived from 16 inter-crossed natural isolates [20]. We used a set of 43 bacterial strains, labeled CeMbio43, as an initial inoculum for the mesocosm experiment and for the common garden experiments. The CeMbio43 community consists of bacterial strains, which were isolated from natural *C. elegans* and its substrate, and which are representative of the native *C. elegans* microbiome (see full list in Supplementary Table S1.1 [17, 18, 21, 22]).

### Mesocosm experiment

**Fig. 1:**
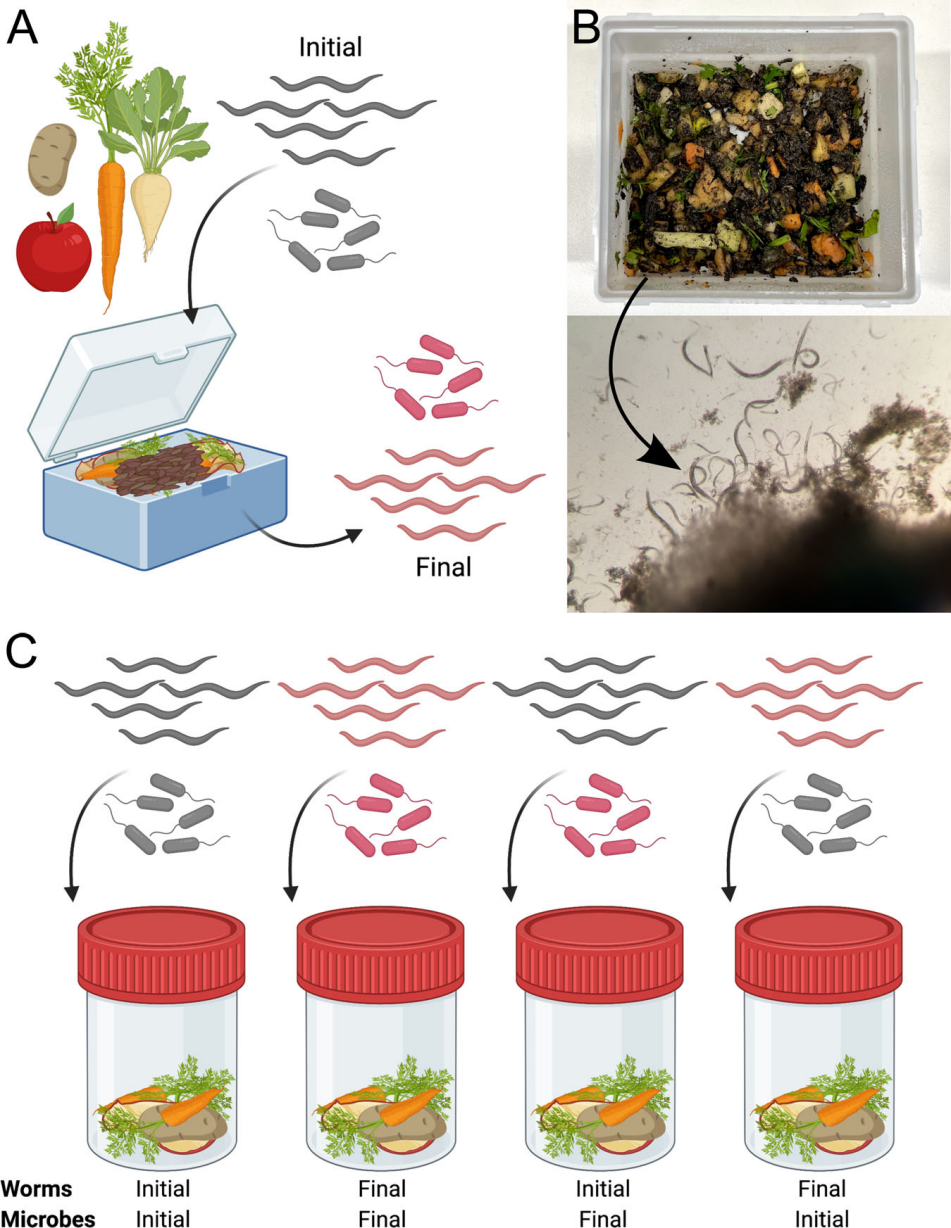
Mesocosm and common garden experiments. **A** For the mesocosm experiment, a genetically diverse, initial *C. elegans* population (initial) and an initial microbiome (initial) were allowed to adapt to a laboratory compost environment. *C. elegans* populations (final) and microbial communities (final) from six mesocosms were isolated at day 100. **B** The initial compost of the mesocosm experiment consisted of compost soil and plant material (top). Worms from proliferating mesocosm populations were isolated from compost samples covered with a buffer (bottom). **C** In a common garden experiment, the initial *C. elegans* population and the initial microbiome were combined with the final nematode populations and the corresponding final microbial communities (from the same mesocosm line) in all possible four combinations in either a compost environment (as illustrated) or an agar plate (not shown). **A**, **C** Created with BioRender.com.

To assess adaptation of the metaorganism, we set up a mesocosm experiment, in which the genetically diverse A_0_ *C. elegans* population and the initial inoculum of the CeMbio43 bacteria were subjected over 100 days to a non-sterile environment consisting of decomposing fruits and vegetables (Fig. 1A; see supplement for more details). This compost environment has not been experienced by the experimental A_0_ population, yet it is related to the natural habitat of *C. elegans* [16, 19], thereby representing a generally suitable context for the worms. See supplementary information for details on the preparation of laboratory compost and collection of worms and bacteria.

### Common garden experiment and assessment of nematode population growth rate

To determine how the host-microbiome assemblage adapted to the new compost environment, we isolated nematode populations and microbial communities from the six mesocosm boxes (aka six independent mesocosm lines) at day 100 and tested them in a common garden experiment. We performed two sets of common garden experiments: the first with material from all mesocosm lines (using only 1 technical replicate), and the second with only material from lines from Box 1 and Box 2 that showed opposite patterns in the first experiment (using five replicates per treatment combination). For each common garden experiment, we combined the initial A_0_ nematode population and the initial microbial inoculum with the final (day-100) nematode populations and the corresponding final (day-100) microbial community (from the same mesocosm replicate) in all possible four combinations in either a compost (both sets of common garden experiments) or on agar plates (only the second), the latter used as an alternative environment known to be suitable for nematode proliferation from the multitude of *C. elegans* studies. Each experiment was followed by an assessment of *C. elegans* population growth (both common garden experiments) and also nematode length, and nematode area (only the second experiment) as proxies for host fitness. Since *C. elegans* inhabits short-lived habitats, which are usually colonized by very small initial populations, rapid population expansion is considered a key trait underlying evolutionary fitness under these habitat conditions [16] and can be assessed by our measure of population growth rate, as done commonly in past studies [22–24]. The body size of *C. elegans* is correlated with fecundity under standard laboratory conditions [25] and may thus serve as an additional, indirect proxy for fitness.

For the second set of common garden experiments, we additionally used the obtained material for an analysis of microbial community composition using both 16S rRNA gene and ITS amplicon sequence analysis (for bacteria and fungi, respectively), and also an analysis of the *C. elegans* transcriptome response. See supplementary information for details on the preparation of laboratory compost, collection of worms and bacteria, and statistics.

### 16S rRNA gene and ITS amplicon sequencing for microbiome analysis of common garden experiment

Microbial community composition was characterized by substrate samples and nematodes, collected at the end of the second set of compost common garden experiments, involving Box 1 and Box 2. After DNA isolation, we used 16S rRNA gene and ITS amplicon sequencing to determine the relative abundance of bacteria and fungi, respectively, following established protocols for sequencing, data processing, and statistical analyses, as outlined in detail in the supplementary information.

### RNAseq for transcriptome analysis of *C. elegans* populations

We assessed the transcriptomic response of *C. elegans* populations from all treatment combinations of the second set of common garden experiments with compost. Compost and worms for transcriptomics were prepared separately but using the same general approach as for the population growth assay in five replicates. Worms were isolated after 24 h, followed by RNA isolation, RNAseq, and transcriptome data analyses, following established protocols [26, 27], as outlined in detail in the supplementary information.

## Results

### Novel compost mesocosm supports stable proliferating *C. elegans* populations

We developed a novel protocol for the long-term maintenance of proliferating populations of *C. elegans* in laboratory compost mesocosms. We exposed a genetically diverse *C. elegans* population to an initial microbiome in laboratory mesocosms consisting of decomposing plant material (i.e., non-sterile chopped vegetables) and soil (Fig. 1A, B; Supplementary Fig. S1). Regular addition of plant material served to supply the mesocosm microbiomes with nutrients and in turn led to consistently high worm counts. Using this protocol, we maintained proliferating worm populations (Fig. 1B; Supplementary Movies 1–3) for more than 600 days (equivalent to approx. 180 *C. elegans* generations under standard laboratory conditions; mesocosm experiment still ongoing), demonstrating that the mesocosm compost provides suitable conditions for stable and continuous growth of *C. elegans* under semi-natural conditions.

### Nematode fitness components in the compost environment are influenced by both host and microbiome

To determine how the nematode and microbial populations responded to the compost environment, we focused on an analysis of worms and microbiomes harvested after 100 days (equivalent to approx. 30 *C. elegans* generations under standard laboratory conditions; Fig. 1B). We co-inoculated different combinations of nematodes and microbiomes into common environments (“common garden” experiments; Fig. 1C). At the conclusion of these experiments, we measured several components of nematode fitness, including population growth rate and nematode size (i.e., length and area; see Methods above).

**Fig. 2:**
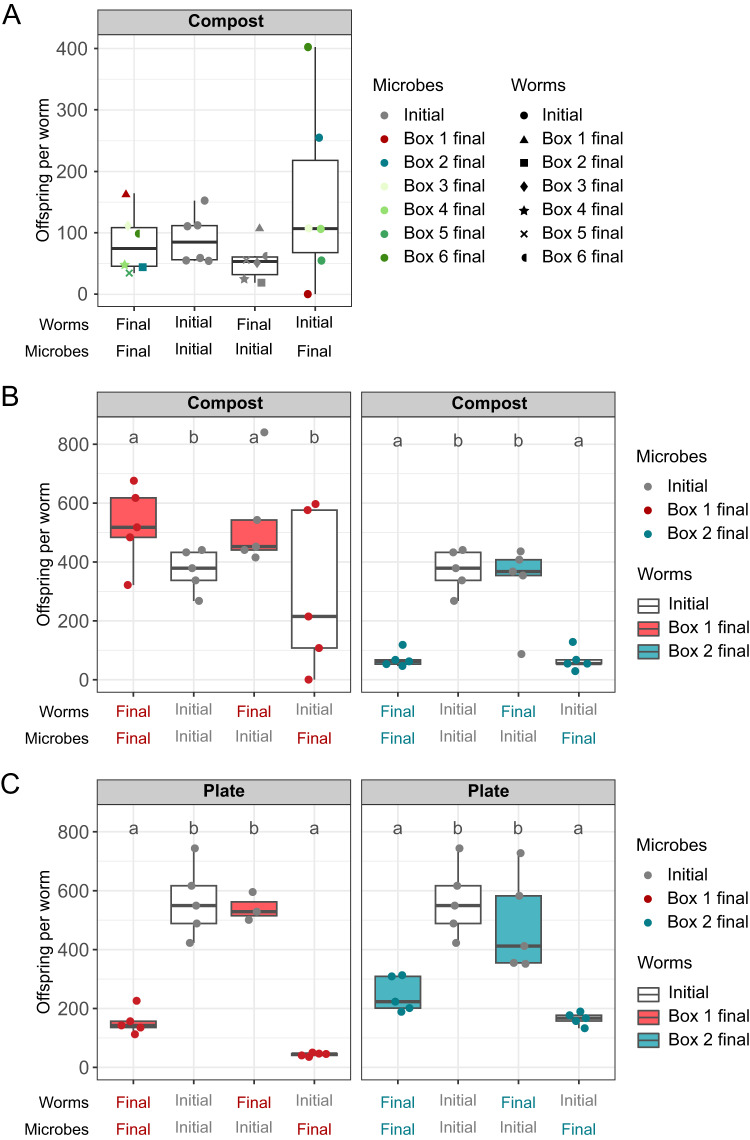
Host and microbiome can jointly determine nematode fitness in the novel compost environment. Results of common garden experiments, in which population growth was measured for *C. elegans* populations isolated from mesocosms at day 100 (final) and initial worms (initial) in the presence of mesocosm day-100 microbiomes (final) or the initial microbiomes including the CeMbio43 bacterial community (initial). Population growth is shown as offspring per worm added at the beginning of the experiment. **A** Population growth of six mesocosm lines (Boxes 1–6) and one initial worm population, measured under compost conditions. Colors indicate different mesocosm microbiomes and the initial microbiomes (gray); symbols indicate worm populations from the different mesocosm boxes and the initial worm population. The combinations of worms and microbes are represented by a combination of a color and symbol of the data points. *n* = 1. **B** Population growth under compost conditions of final Box 1 (red boxes), final Box 2 (blue boxes), and initial worm populations (white boxes) in the presence of final Box 1 (red dots) or final Box 2 (blue dots) microbiomes or initial microbiomes (gray dots). *n* = 5. **C** Population growth on agar plates of final Box 1 (red boxes), final Box 2 (blue boxes), and initial worms (white boxes) with final Box 1 (red dots), final Box 2 (blue dots) or initial microbiomes (gray dots). Results are summarized as boxplots with the median as a thick horizontal line, the interquartile range as box, the whiskers as vertical lines, and each replicate depicted by a dot or symbol. Significant differences are indicated with different letters. *n* = 5. Note that the replicates for initial worms with the initial microbiome were used for both, the Box 1 and Box 2 treatments in 2B and 2C.

Our first common garden experiment consisted of inoculating final mesocosm worm populations or initial worm populations into fresh compost, either with the corresponding final microbiome (from the same mesocosm line) or with the initial microbiome used for the initial inoculation of the mesocosms. Overall, measures of worm fitness did not differ between final worms and initial worms (Fig. 2A; Supplementary Tables S1.2 and S1.3). However, we observed substantial differences among the independent mesocosm lines, particularly when combined with different microbiomes. We chose two exemplary mesocosm lines with contrasting patterns, labeled Box 1 and Box 2, for subsequent analyses (Fig. 2A). During this first common garden experiment, the final Box 1 worms produced high numbers of offspring, especially when inoculated with their respective final microbiomes, whereas initial worms produced almost no offspring with the same microbial inoculum. In contrast, Box 2 final worms produced few offspring when inoculated with their final microbiomes or with the initial microbiome, whereas initial worms produced many offspring when inoculated with these same microbiomes. These results suggest that the relative contribution of host and microbiome to metaorganism fitness may have diverged substantially between Box 1 and Box 2 over the 100 days of this experiment. However, this conclusion is based on single replicates of each combination of worm population and microbiome.

In the second set of common garden experiments we asked whether this conclusion was robust to replication. These experiments focused only on the Box 1 and Box 2 worm populations and microbiomes (excluding those from the other mesocosms). We assessed the considered fitness components of the final worm populations and the initial worm population, each combined with either final microbiomes or the initial microbiome. We conducted these experiments in a compost environment (as before) and additionally on agar plates, in order to determine whether changes in fitness were specific to the compost environment.

In Box 1 compost, the number of worm offspring produced was significantly influenced by the nature of the worm population (i.e., final vs initial; *p* = 0.027; Supplementary Tables S1.4, S1.5), whereas the size of individual worms (i.e., worm area) was affected by the type of microbial inoculum (i.e., final vs initial microbiomes; *p* = 0.015; Supplementary Fig. S2; Supplementary Tables S1.6–S1.8). As in the first common garden experiment, the final Box 1 worms produced many offspring when combined with their final microbiome (Fig. 2B). The number of offspring of the initial worms inoculated with the final Box 1 microbiome varied substantially among replicates (Fig. 2B). In contrast, the final Box 2 microbial inoculum consistently and significantly reduced worm offspring numbers, worm length, and worm area produced by both the final Box 2 and the initial worms (in all cases), *p* < 0.01; Fig. 2B; Supplementary Tables S1.4-S1.8 Taken together, under compost conditions and thus the relevant conditions of the earlier mesocosm experiment, the final Box 1 nematode population has increased in a relevant component of nematode fitness, worm population growth rate, while we did not observe any fitness change for the final Box 2 worm population. Furthermore, the results suggest that the final Box 1 and final Box 2 microbiomes influence components of nematode fitness.

On agar plates, the microbial inoculum significantly influenced the offspring numbers, worm length, and worm area (*p* < 0.01; Fig. 2C; Supplementary Fig. S2; Supplementary Tables S1.9–S1.13). For Box 1 worms, the results on agar differed from those in compost; offspring numbers were higher on plates containing the initial microbiome than those with final Box 1 microbiomes, and initial worms produced the fewest offspring when inoculated with final Box 1 microbiomes (Fig. 2C). For Box-2 worm populations, the number of offspring per worm was significantly influenced by the type of the microbial inoculum (*p* < 0.001; Supplementary Tables S1.9, S1.10), similar to the results observed in compost (Fig. 2B, C). It is worth noting that both measures of worm size were correlated with worm offspring numbers in compost as well as on agar plates (Supplementary Fig. S2), supporting the previous observation [25] that both are related and that worm size represents a meaningful proxy for fitness. Overall, these results suggest that at least the changes in Box 1 population growth were specific to the compost environment and were influenced by changes in both the host population and the microbiome.

### Compost and nematode microbiomes differ by inoculum source

We explored which changes in microbial communities underlie the observed changes in fitness components in the second common garden experiment with host and microbes from Box 1 and Box 2. Bacterial communities in the common garden experiments were dominated by a combination of genera present in the CeMbio43 inoculum (including *Acinetobacter*, *Pseudomonas*, *Gluconobacter*, and *Stenotrophomonas* of the phylum Proteobacteria and *Sphingobacterium* of the phylum Bacteriodota) and new genera likely introduced via plant material added to the mesocosms (including *Leuconostoc*, *Lactococcus*, and *Anaerosporobacter* of the phylum Firmicutes; *Dysgonomonas* and *Bacteroides* of the phylum Bacteriodota; and *Leucobacter* of the phylum Actinobacteriota). As there were no fungal taxa present in the CeMbio43 inoculum, all fungi detected in the microbiome samples were introduced via added plant material. The most abundant genera in the fungal communities were *Candida*, *Barnettozyma*, *Hanseniaspora*, and *Pichia* which belong to the Ascomycota.

**Fig. 3:**
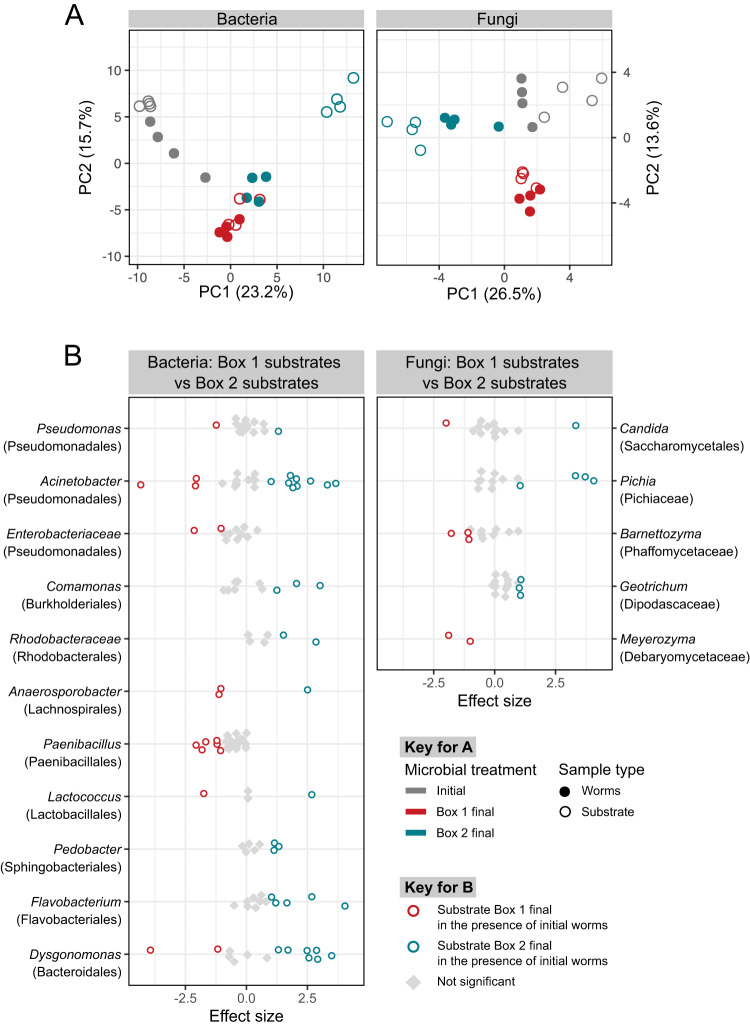
Inoculum source influenced both compost and nematode microbiomes. **A** Ordination (Principal Coordinates analysis of Aitchison distance) depicting variation in microbiome composition across inoculation treatments in a common garden experiment. Colors indicate microbial treatment using final microbes from Box 1 (red) or Box 2 (blue), or initial microbes (gray); filled circles indicate sample type worms, open circles indicate sample type substrate (compost with associated worms). Each symbol represents one replicate. *n* = 4 **B** Differential abundance analysis of substrate microbiomes inoculated with final Box 1 microbiome or final Box 2 microbiome. Each point represents an ASV and ASVs are grouped by genera with family listed in parentheses. Genera with at least two differentially abundant ASVs are included here (see Supplementary Figs. S3 and S4 for complete results). Colors indicate ASVs with significantly greater relative abundance in either substrate inoculated with the final Box 1 microbiome (red) or substrates inoculated with the final Box 2 microbiome (blue).

To determine whether the final mesocosm lines from Box 1 and Box 2 varied in microbiome composition, we focused on the common garden experiments in which initial worms were exposed to three distinct inocula (i.e., final Box 1 microbiomes, final Box 2 microbiomes or the initial microbiomes) in the compost environment. Inoculum source was the strongest influence on microbiome composition, across both worm and substrate samples, explaining over 30% of the variation among these samples for both the bacteria and fungi (Fig. 3A; Supplementary Tables S2.1–S2.3). Worm and substrate samples also differed from each other in microbiome composition; however, this depended on the mesocosm line. Specifically, worm and substrate microbiomes differed significantly when initial worms were exposed to the initial microbiome (16S rRNA gene, *R*^2^ = 0.25, *p* = 0.03; ITS, *R*^2^ = 0.29, *p* = 0.03) or final Box 2 microbiomes (16 S rRNA gene, *R*^2^ = 0.42, *p* = 0.04; ITS, *R*^2^ = 0.27, *p* = 0.03) but not the final Box 1 microbiomes (16S rRNA gene, *R*^2^ = 0.18, *p* = 0.14; ITS, *R*^2^ = 0.11, *p* = 0.74). These results suggest that the substrate microbiome from the Box 1 treatment contains microbes that are able to colonize *C. elegans*, are preferentially taken up by the nematodes, and/or are able to resist colonization by microorganisms from added fruits and vegetables. In contrast, the Box 2 microbiome treatment results in significantly different substrate and worm microbiomes, indicating the presence of microbes that cannot colonize nematodes and/or are avoided by *C. elegans*.

We investigated the specific taxonomic differences between the Box 1 and Box 2 substrate microbiomes and identified both bacterial and fungal taxa (i.e., amplicon sequence variants or ASVs) with differential abundance (Fig. 3B). For example, ASVs from the fungal genera *Pichia* and *Geotrichum* and the bacterial genera *Comamonas*, *Pedobacter*, and *Flavobacterium* were more abundant in substrates receiving the final Box 2 inoculum. Substrates receiving the final Box 1 inoculum were enriched in ASVs from the fungal genera *Barnettozyma* and *Meyerozyma*, and the bacterial genera *Paenibacillus* and *Anaerosporobacter* (Fig. 3B). A subset of these taxa was differentially abundant between initial worms exposed to the final Box 1 inoculum and those exposed to the final Box 2 inoculum (Supplementary Figs S3, S4).

### Microbiome community changes are associated with differences in nematode fitness components

**Fig. 4:**
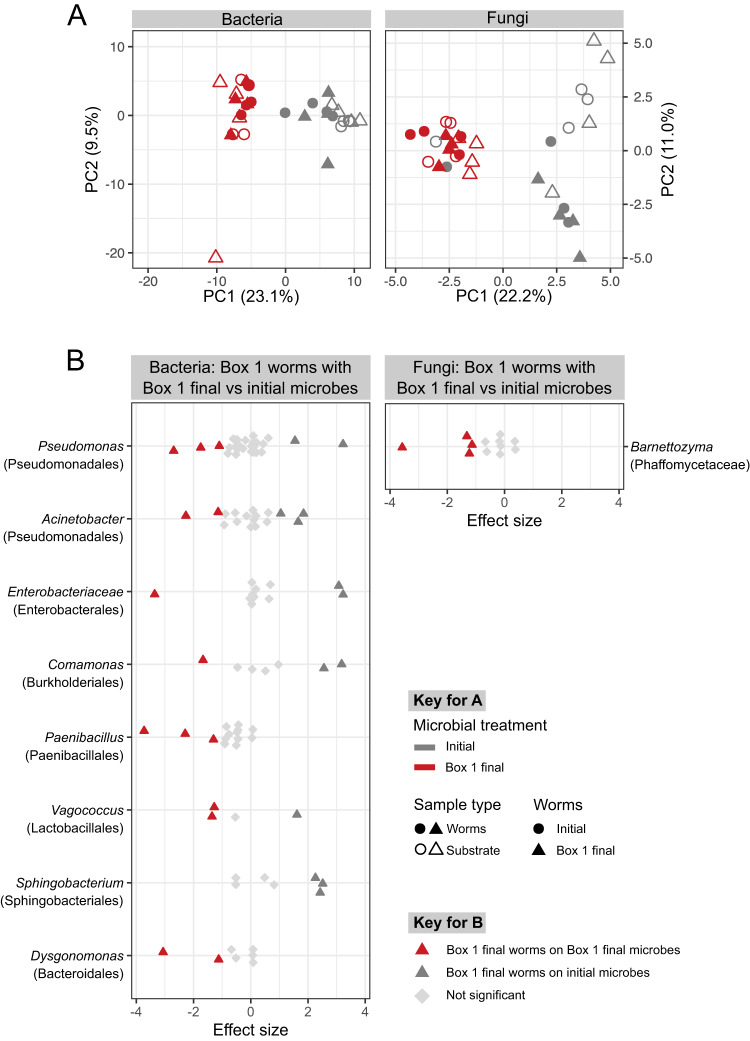
Differences in microbiome composition were associated with increased fitness in nematodes from the Box 1 mesocosm. **A** Ordination (Principal Coordinates analysis of Aitchison distance) depicting variation in microbiome composition across treatments in a common garden experiment. Colors indicate microbial treatment using final microbes from Box 1 (red) or initial microbes (gray); Symbols indicate initial (circle) or final Box 1 (triangle) worm origin; filled symbols indicate worm samples, open symbols indicate substrate samples (compost with associated worms). *n* = 4. **B** Differential abundance analysis of final microbiomes from Box 1 worms exposed to the final Box 1 microbiome or the initial microbiome. Each point represents an ASV and ASVs are grouped by genera with family listed in parentheses. Genera with at least two differentially abundant ASVs are included here (see Supplementary Figs. S5 and S6 for complete results). Colors indicate ASVs with significantly greater relative abundance in either worms inoculated with the final Box 1 microbiome (red) or worms inoculated with the initial microbiome (dark gray).

We focused on understanding the microbiome contributions to the increased fitness observed for final Box 1 worms. To accomplish this, we compared treatments with either initial worms or final Box 1 worms in combination with either the initial microbiome inoculum or the final Box 1 inoculum. Across these treatments microbiome composition differed significantly between inocula type for both the substrate and worms. Worm population source did not influence the microbiome composition of either worms or substrate samples (Fig. 4A; Supplementary Table S2.4). Microbiome composition was very similar between worm and substrate samples in those treatments that received the final Box 1 inoculum. Worm and substrate microbiomes were also similar in those treatments that received the initial inoculum, but to a lesser degree. Variation among replicates was smaller for worms exposed to the final Box 1 inoculum compared to the initial inoculum. This pattern was consistent for both fungal and bacteria microbiomes (Fig. 4A; Supplementary Tables S2.1, S2.2, S2.4). These results suggest that the final Box 1 substrate microbiomes are consistently associated with *C. elegans*, irrespective of worm source. To identify microbes that may contribute to the higher population growth rate observed for the final Box 1 worms exposed to final Box 1 microbiome, we looked for ASVs that were differentially abundant in final Box 1 worms exposed to either the co-existing final Box 1 microbiome or the initial microbiome. Those worms exposed to the final Box 1 microbiome had a consistently higher relative abundance of ASVs from the fungal genus *Barnettozyma* and bacterial genera *Paenibacillus* and *Dysgonomonas*, and a lower relative abundance of *Sphingobacterium* (Fig. 4B). Some bacterial genera (e.g., *Pseudomonas* and *Acinetobacter*) exhibited an inconsistent response, with some ASVs within the genus being more and others less abundant in worms with the final Box 1 inoculum. This result suggests that differences among microbial species or even strains may have important consequences for the increased population growth rate we observed. When worms exposed to the final Box 1 inoculum were compared to their respective substrates, we observed few differentially abundant ASVs and no consistent taxonomic differences between the sample types (Supplementary Figs. S5, S6).

**Fig. 5:**
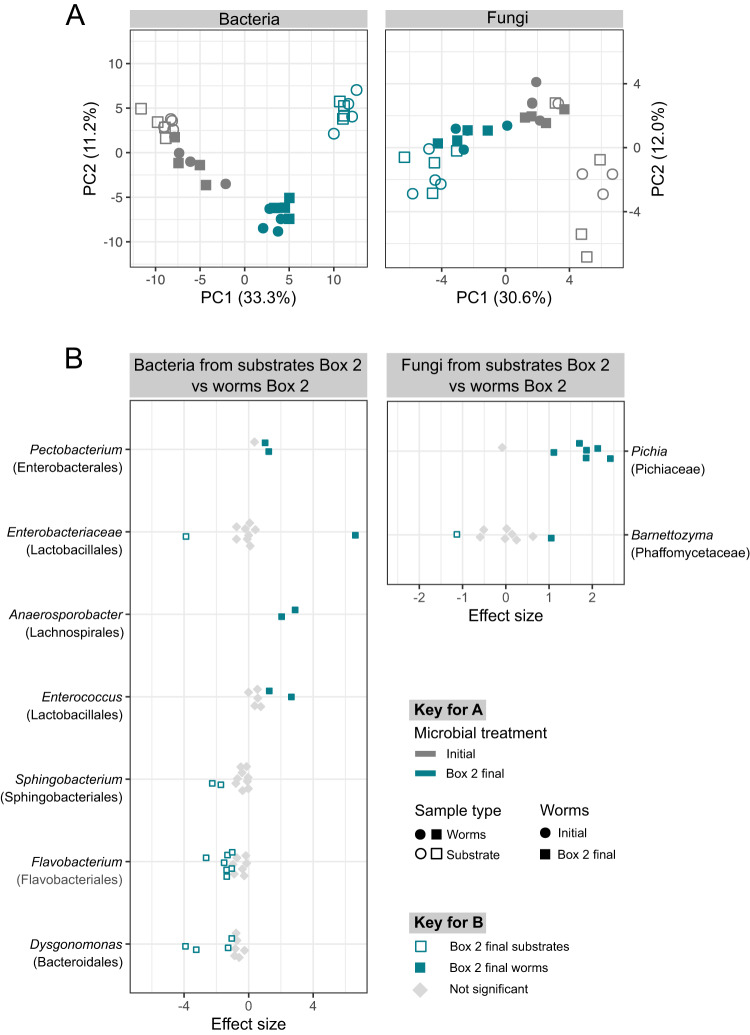
Differences in microbiome composition were associated with decreased fitness in nematodes from the Box 2 mesocosm. **A** Ordination (Principal Coordinates analysis of Aitchison distance) depicting variation in microbiome composition across treatments in a common garden experiment. Colors indicate microbial treatment using final microbes from Box 2 (blue) or initial microbes (gray); Symbols indicate initial worm origin (circle) or final Box 2 (square) worm origin; filled symbols indicate worm samples, open symbols indicate substrate samples (compost with associated worms). *n* = 4 **B** Differential abundance analysis of substrate and worm microbiomes exposed to the final Box 2 microbiome. Each point represents an ASV and ASVs are grouped by genera with family listed in parentheses. Genera with at least two differentially abundant ASVs are included here (see Supplementary Figs. S7 and S8 for complete results). Shapes indicate ASVs with significantly greater relative abundance in either substrate (open) or worms (filled) inoculated with the final Box 2 microbiome.

To identify microbiome contributions to the decreased worm population growth rate associated with the final Box 2 microbiome, we compared treatments with either initial worms or worms from the final Box 2 mesocosms in combination with the initial inoculum or microorganisms from the final Box 2 mesocosms. Microbiome composition differed significantly between inoculum types for worm and substrate samples, and there was no influence of worm population source on microbiome composition, similar to what was observed in the analysis of the Box 1 treatment combinations. However, while worm and substrate samples tended to be similar (i.e., cluster together; Fig. 4A) for the Box 1 treatment combinations, we observed significant separation of worm and substrate microbiomes when exposed to the microbes from the final Box 2 mesocosms (Fig. 5A, Supplementary Tables S2.1, S2.2, S2.5). This result suggests that overall the final Box 2 substrate microbiome does not associate with the host nematode, regardless of the evolutionary history of the nematode. To determine which microbial taxa underlie this separation, we compared microbiomes of worms and substrate samples from the final Box 2 microbiome treatments. Relative to worms, substrates had higher relative abundance of ASVs from the bacterial genera *Sphingobacterium*, *Flavobacterium*, and *Dysgonomonas*. ASVs from the bacterial genera *Pectobacterium*, *Anaerosporobacter*, and *Enterococcus* and the fungal genera *Pichia* had a higher relative abundance in the worm samples relative to the substrate samples (Fig. 5B). Most of these same enriched taxa were observed regardless of whether initial worms or final Box 2 worms were compared to substrate (Supplementary Figs. S7, S8).

### Gene expression differs between different nematode populations inoculated with the same microbiomes

It is clear from our common garden experiments that the Box 1 nematode population increased in the considered fitness component population growth rate following 100 days in the laboratory compost environment and that additionally the final Box 1 microbiome produces a compost-dependent increase in population growth rate. It is also clear that the Box 2 microbiome caused a decrease in the considered fitness proxies. To determine whether these changes in fitness components were also associated with changes in the host population, we compared variation in gene expression among the Box 1, Box 2, and initial *C. elegans* populations after exposure to the initial microbiome in the compost environment. Assessing gene expression in a common environment and after exposure to identical microbiomes allowed us to isolate host genetic changes from those induced by environmental differences.

**Fig. 6:**
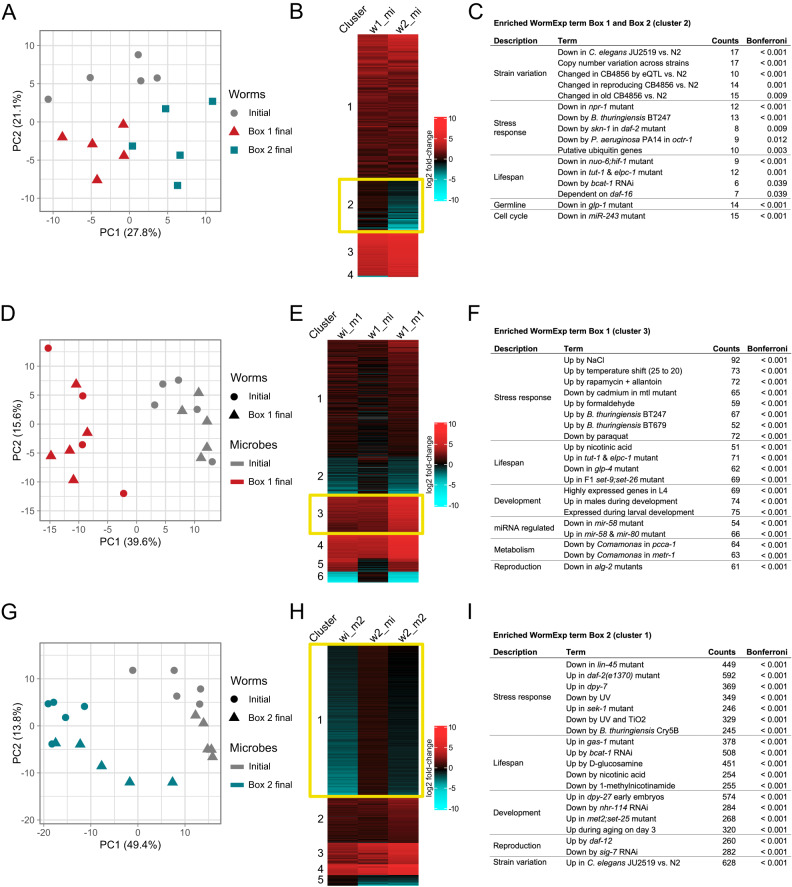
Differential gene expression in the adapted Box 1 and Box 2 *C. elegans* populations. Transcriptome data analysis for the three main sets of analyses, including **A**–**C** a comparison of initial, final Box 1, and final Box 2*C. elegans* populations assayed under identical compost conditions with the initial microbiomes including the CeMbio43 bacterial community, **D**–**F** a comparison of all possible host-microbiome combinations for the Box 1 common garden experiment, and **G–I** a comparison of all possible host-microbiome combinations for the Box 2 common garden experiment. In the latter two cases, initial or final Box 1/Box 2 worms were combined with either the initial microbiomes or the final Box 1/Box 2 microbiomes. In all three cases, general variation in gene expression was explored with a principal component analysis, whereby panels **A**, **D**, **G** show the spread of sample variation along the first two principal components (PC1, PC2). Symbols indicate final Box 1 (triangle), final Box 2 (square), or initial (circle) worm origin; colors indicate final Box 1 (red), final Box 2 (blue), or initial (gray) microbiome origin. *n* = 5. Thereafter, variation in significantly differentially expressed genes was assessed using *k*-means clustering and visualization of differential expression using heatmaps, whereby the heatmaps always show fold change of gene expression relative to the initial worms combined with initial microbiomes (**B**, **E**, **H**). The clusters, which were used for a focused enrichment analysis, are highlighted by a yellow rectangle. Abbreviations: wi, initial worm population; w1, final Box 1 worm population; w2, final Box 2 worm population; mi, initial microbiome; m1, final Box 1 microbiome, m2, final Box 2 microbiome. **C**, **F**, **I** show the results of the enrichment analysis with the *C. elegans*-tailored WormExp database. Description in the first column indicates overarching functions for the enriched gene sets, for which terms are given in the second column. The third column shows the number of genes of the query set that are responsible for the enrichment with the indicated gene set, while the fourth column shows the inferred probability of enrichment, after Bonferroni correction.

An explorative PCA revealed a clear separation of the final Box 1 and Box 2 worm populations along the first principal component (PC1, explaining 27.8% of the variation), whereas the initial population diverged from the two others along PC2 (21.1% variation; Fig. 6A; Supplementary Fig. S9B). A subsequent *k-means* clustering of the significantly differentially expressed genes yielded four distinct clusters, of which clusters 2 and 4 indicated genes that show contrasting expression patterns for final Box 1 and Box 2 nematode populations (Fig. 6B; Supplementary Table S3.1). Cluster 4 consisted of a single gene, *srh-178*, encoding a transmembrane G protein-coupled receptor belonging to the class H serpentine receptors with no specific known function, that is upregulated in the final Box 2 but downregulated in the final Box 1 worms. Cluster 2 included 41 genes, which we used for a focused enrichment analysis. While the DAVID analysis did not indicate any clearly enriched categories (Supplementary Fig. S9A), the *C. elegans*-tailored gene expression analysis with WormExp showed an overrepresentation of gene sets with few main functions, including genes known to be differentially regulated among different natural *C. elegans* strains (i.e., enriched category “Strain variation”, Fig. 6C) and additionally stress response as well as lifespan genes, which are generally downregulated in the final Box 2 but not final Box 1 worms (Fig. 6C; Supplementary Fig. S9C; Supplementary Table S3.2). These results provide a strong indication that the two *C. elegans* populations did indeed change genetically and thus evolved in their mesocosms. Moreover, the results indicate that the final Box 1 and Box 2 populations diverged from each other, whereby the final Box 2 worms showed a reduction in expression of stress response and lifespan genes under these standardized conditions. Subsequently, we asked whether each of the separate common garden experiments indicates a host transcriptome response that is consistent with the observed phenotypic variation.

### Gene expression differs between identical nematode populations inoculated with different microbiomes

To determine whether microbiome composition influenced host gene expression, we compared variation in gene expression among the final Box 1, final Box 2, and initial *C. elegans* populations after exposure to different microbiomes in the compost environment. For Box 1, we compared the gene expression of the final nematode population or the initial population inoculated with either the final Box 1 microbiome or the initial microbiome in a full factorial design. The initial explorative PCA indicated a clear separation by microbiome type along PC1 (Fig. 6D, explaining 36.9% variation). Worm populations (final Box 1 vs initial) were separated along PC3 (Supplementary Fig. 10B, explaining 9.7% variation). Subsequent *k*-means clustering identified six distinct clusters (Fig. 6E; Supplementary Table S3.3). Of these, cluster 3 produced the most convincing pattern of a distinct expression profile for final Box 1 worms exposed to final Box 1 microbiomes in comparison to all others, in this case consisting of a pronounced upregulation of 88 genes. To specifically explore which possible gene expression functions account for the high performance of the Box 1 worms with Box 1 microbiomes, we focused the enrichment analyses on only this cluster 3. The DAVID analysis revealed an enrichment of the GO terms pseudopodium, carbohydrate binding, cytoskeleton, and cytoplasm, all with medium importance (Supplementary Fig. 10A). The WormExp analysis further identified an enrichment of numerous gene sets, including the strong upregulation of genes involved in stress responses, lifespan extension, and development (Fig. 6F; Supplementary Fig. 10C; Supplementary Table S3.4). The indicated upregulation of stress responses is supported by several enriched gene sets, including for example gene sets upregulated upon exposure to NaCl, cadmium, or paraquat. Interestingly, these gene sets differ from the enriched gene sets, which similarly contribute to stress responses and which were differentially regulated between the different worm populations (final Box 1 worms *versus* final Box 2 worms; Fig. 6C). These results strongly indicate that the high population growth rate of Box 1 worms colonized with Box 1 microbes in the compost environment is associated with relatively higher expression of genes involved in stress responses that target distinct abiotic as well as biotic stresses (e.g., osmotic stress, heavy metals, toxic substances, and pathogens, Fig. 6F; Supplementary Table S3.4). In addition, high population growth rate under these conditions is associated with upregulation of longevity and developmental genes.

For Box 2, we again used a full factorial design and compared the gene expression of either the final or initial nematode population inoculated with either the final Box 2 microbiome or the initial microbiome. We observed that inoculation with the final Box 2 microbiome resulted in the downregulation of distinct stress responses in both the final Box 2 as well as the initial *C. elegans* populations. Exposure to the final Box 2 microbiomes produces a clearly distinct gene expression in both host populations, as revealed by the explorative PCA (Fig. 6G; Supplementary Fig. 11B) and also the differential gene expression analysis, indicated especially by cluster 1 of the *k*-means clustering analysis (Fig. 6H; Supplementary Table S3.5). This cluster 1 includes downregulation of 782 genes. Using DAVID, cluster 1 is enriched for a large number of GO terms, especially those for regulation of transcription and DNA binding (Supplementary Fig. 11A). The WormExp analysis similarly revealed enrichment of numerous gene sets (Fig. 6I; Supplementary Fig. 11C; Supplementary Table S3.6), ultimately indicating the downregulation of genes involved in distinct stress responses (e.g., UV and pathogen stress), lifespan, development, and reproduction. The indicated downregulation of stress responses is supported by enriched gene sets that are again distinct from the gene sets, which we found above to underlie the upregulated stress responses in Box 1 worm populations, indicating differences in the types of stress responses differentially regulated by final Box 1 worms or final Box 2 microbiomes. Overall, these results suggest that the microbial community from Box 2 compromises nematode stress responses, most likely explaining the observed decrease in worm fitness, irrespective of *C. elegans* population.

## Discussion

We established a novel experimental metaorganism model that enables the long-term cultivation of *C. elegans* populations and the assessment of metaorganism adaptation to a complex environment. To date, *C. elegans* has been used for numerous experimental evolution studies [28], focused for example on the assessment of mutation accumulation under relaxed selection [29], adaptation to fluctuating environments [30], or host-pathogen coevolution [31, 32]. All of these studies have been performed under highly artificial laboratory conditions, where worms are maintained on agar plates or in a standardized liquid medium, usually supplemented with a single food bacterium, *Escherichia coli* OP50, which it does not encounter in nature [16]. In all of these studies, *C. elegans* is maintained without its microbiome, which is removed via a bleaching protocol that kills bacteria but not nematode eggs. The model we describe here permits analysis of *C. elegans* with its microbiome in a structured compost environment similar to its natural habitat [16, 19]. A compost environment was used previously for *C. elegans*-microbiome studies, but only for short-term experiments [33]. In our model, the compost environment was inoculated with an initial microbiome including the CeMbio43 bacterial community, a representative mixture of bacteria from the native microbiome of *C. elegans* (Supplementary Table 1.1; [18, 22]). Since the mesocosm was by design maintained under non-sterile conditions, additional microorganisms colonized the mesocosms in addition to this mixture. This set-up allowed us to explore how the *C. elegans* metaorganism adapted to the compost environment.

We observed that adaptation took different trajectories in different mesocosm lines over 100 days, with some increasing in a naturally relevant component of nematode fitness, worm population growth rate, and others decreasing. This is unexpected, given that all of the replicate mesocosms were initiated with the same worm population, the same initial bacterial inoculum and the same starting plant material, and were maintained under identical conditions. We do not know in detail what caused these different trajectories, but they could be driven by random mutation events in the host genome, stochastic microbiome assembly across the mesocosm lines, and/or subtle differences among the lines in starting conditions, among many possible causes.

We identified one exemplary test case for further study, mesocosm Box 1, which strongly increased in the considered fitness component worm population growth (Fig. 2B). We obtained a strong indication for genetic changes in the *C. elegans* population: (i) The final Box 1 nematode population differed significantly in population growth from the initial nematode population (Fig. 2B), which is most likely caused by genetic changes but not by plastic or epigenetic effects of the Box 1 worms because these were processed for at least 4 generations outside the compost upon isolation from the mesocosm Box 1 (including bleaching of worms, freezing of worms, thawing and subsequent growth of worms). (ii) The final Box 1 and the initial worm populations differed significantly in gene expression variation measured under otherwise completely identical conditions (i.e., upon combination with the initial CeMbio43 microbiome), as visualized in the PCA plot in Fig. 6A, and as further supported by the top significantly enriched WormExp gene sets, many of which refer to gene expression variation previously observed between different natural *C. elegans* strains (Fig. 6C). Overall, the host appears to have evolved by increasing its general stress response (Fig. 6C, F), possibly allowing it to cope well with a complex compost environment that is much more structurally and physiologically challenging than the agar plate environment it previously experienced.

The results from the Box 1 common garden experiment suggest that these host genetic changes lead to increased worm population growth rate irrespective of the microbiome inoculum (Fig. 2B). Nevertheless, the coexisting microbiome from the Box 1 mesocosm has a highly specific influence on population growth rate of the adapted worm population from Box 1, because it only causes an increase in population growth in the compost environment but not on agar plates, leading to the highest median fitness values in compost (Figs. 2B, 2C). Thus, our study provides one of the first experimental demonstrations of a joint host genetic and microbiome effect on metaorganism adaptation. Most previous work focused on the contribution of the microbiome or host genome alone to adaptation (discussed in [9, 34]), with the exception of the recent study in *Nasonia* wasps, for which metaorganism adaptation to the herbicide atrazine was associated with changes in both host genetics and microbiome composition [15].

We identified both bacterial and fungal lineages associated with this increase in the considered fitness proxy, including lineages that contain microbial species known to be beneficial to *C. elegans*; for example, bacteria from the genus *Pseudomonas* isolated from natural populations of *C. elegans* have been shown to have beneficial effects [17, 23, 24]. There may have been specific evolutionary adaptations in individual microbial lineages that resulted in their positive impact on the fitness of the worm populations that evolved in Box 1; however, this remains a topic for future research since we cannot determine this solely from our amplicon data.

Although we do not yet know exactly how these microbial taxa from Box 1 influence worm fitness, we do have evidence that the final Box 1 microbiome as a whole is well-adapted to association with the *C. elegans* population that evolved in the Box 1 mesocosm, because the compositions of the substrate microbiomes and worm microbiomes resulting from inoculation with microbiomes from the Box 1 mesocosm are very similar to each other (Fig. 3A). This is not the case for other worm and inoculum combinations, which result in worm and substrate microbiomes that are less similar or in some cases substantially different (Fig. 3A). This pattern is consistently present for both fungi and bacteria.

Adaptation through changes to both the microbiome and host are only one of the outcomes that we observed in our experimental system. We also observed a maladaptive response (i.e., a decrease in the measured proxy for metaorganism fitness). We chose one mesocosm that exemplifies this response, Box 2, for further study. The worms from this mesocosm line exhibited decreased fitness in the compost environment, but only when inoculated with the final substrate microbiome from the Box 2 mesocosm. This inoculant caused substantially decreased fitness in the initial worm population as well. It also resulted in a fitness reduction in the agar plate environment for both the initial and the final Box 2 worm population, although not to the same degree as in compost. These results suggest that the microbiome that developed in this mesocosm was generally detrimental to worm health.

We identified both bacterial and fungal lineages associated with this detrimental effect on fitness, which included members of the bacterial genera *Flavobacterium* and *Dysgonomonas*, and the fungal genus *Pichia*. Although we do not yet know how these microbes decrease metaorganism fitness, we do have evidence that the Box 2 microbiome is not well-adapted overall to association with *C. elegans*, because the compositions of the worm microbiomes and substrate microbiomes resulting from inoculation with microbiomes from the Box 2 mesocosm are very different from each other (Fig. 3A). This is true for both fungi and bacteria. This is in contrast to the situation that results from inoculation with the Box 1 microbiome, as described above. This pattern is consistent with the hypothesis that the Box 2 microbiome contains members that are poorly adapted to association with the worm host.

Using our novel *C. elegans*/compost system, we have established that changes in both host and microbiome can jointly mediate metaorganism adaptation. Adaptive evolution is often based on quantitative genetic changes, yet to date, it is unknown how precisely the associated microbiome interacts with host genetics to determine fitness. Our experimental system is sufficiently tractable that it will be possible, in future work, to quantify the relative contributions of both host and microbiome to the adaptive process.

### Supplementary information

Supplementary Information 1

Supplementary Information 2

Supplementary Table S1

Supplementary Table S2

Supplementary Table S3

Supplementary Movie 1

Supplementary Movie 2

Supplementary Movie 3

## Data Availability

The datasets generated during and/or analyzed during the current study are available in Supplementary Tables S1 and S2. The nucleotide sequence data reported are available from the NCBI BioProject database under the BioProject ID PRJNA954426. The raw data for the transcriptomic analysis is available from the ENA database under the accession numbers ERR11455018- ERR11455037.
