# Supplementary material for: Host and microbiome jointly contribute to environmental adaptation
Source: ISME J. 2023 Sep 6;17(11):1953–65. doi: 10.1038/s41396-023-01507-9 (PMC10579302; doi:10.1038/s41396-023-01507-9)
Supplement: Supplementary file 1 — Supplementary Information 1 [file 41396_2023_1507_MOESM1_ESM.pdf]

Supplementary Information

Host and microbiome jointly contribute to environmental adaptation

Carola Petersen[1*], Inga K. Hamerich[1*], Karen L. Adair[2*], Hanne Griem-Krey[1], Montserrat Torres Oliva[3], Marc P. Hoeppner[3], Brendan J.M. Bohannan[2†], Hinrich Schulenburg[1,4 †]

[*]Shared first authors

[†]Shared senior authors

Corresponding author:

h.schulenburg@zoologie.uni-kiel.de

[1] Department of Evolutionary Ecology and Genetics, Kiel University, Kiel, Germany

[2] Institute of Ecology and Evolution, University of Oregon, Eugene, OR, USA

[3] Institute of Clinical Molecular Biology, Kiel University, Kiel, Germany

[4] Max-Planck Institute for Evolutionary Biology, Ploen, Germany

Supplementary Materials and Methods

Nematode strains

We used the *C. elegans* population A_0 (1) which was repeatedly used in various evolution experiments (1–4). Unless otherwise stated, the A_0 population was maintained on nematode growth medium (NGM) plates with *Escherichia coli* strain OP50 and synchronized by bleaching following standard procedures (5).

Bacterial strains

To prepare the CeMbio43 community, bacteria were thawed from frozen stocks, individual bacteria were cultured in tryptic soy broth (TSB) in deep well microtiter plates for 42 h on a circular shaker at 20 °C, then mixed in equal cell numbers, and the bacterial mixture adjusted to OD_{600}10 in PBS.

Mesocosm experiment

Lab compost was prepared by placing 100 g autoclaved compost soil in a sterile box. Each box was closed with a lid with three 3 cm holes closed with a sterile foam plug to allow aeration. Potatoes, apples, kohlrabi, and carrots including leaves were washed with water, chopped, mixed, and approximately 100 g of the plant material was added to each box and mixed with the soil in the box. One ml of freshly prepared CeMbio43 in OD_{600}5 was added directly, and another 1 ml and 1.3 ml were added after 24 h and 48 h, respectively. Compost and bacteria were mixed and moistened with 1 to 2 ml of sterile water. Subsequently, approximately 3200 *C. elegans* A_0 in different stages were added to the compost. These worms were previously maintained on NGM plates inoculated with OP50 and repeatedly washed with M9-buffer prior to introduction into the mesocosm boxes. The boxes were stored on a table in a separate room at room temperature (seasonal fluctuations between 17 °C and 25 °C). Fresh plant material was added every other week and each compost mixed every week using a sterile wooden spatula. The position of the boxes on the table was shuffled weekly. In order to mimic a natural compost environment, the boxes were not maintained under strictly sterile conditions; therefore, microorganisms (in addition to the initial CeMbio43 inoculum) were regularly introduced to the environment (e.g. via fresh plant material).
After 100 days, we sampled nematodes and bacteria from each box. To collect the microbial communities, a randomly chosen subsample of each compost was placed in a 9-cm petri dish, followed by addition of 25 ml of M9-buffer with 0.025% (v/v) of Triton X100 (M9-T). We transferred 300 µl of the mixture to a 2 ml microtube containing approximately ten sterile 1 mm zirconia beads, followed by homogenization of the material using a bead ruptor (Bead Ruptor 96, Omni International, Kennesaw, Georgia, USA) for 3 min at 30 Hz, and conservation of microbes in 10% (v/v) dimethyl sulfoxide (DMSO) at -80 °C. To obtain microbe-free nematodes, we transferred 150 µl of worm-containing buffer from the same compost

subsample onto peptone-free NGM (PFM) plates. The resulting worm population was bleached to remove any bacteria, following standard protocols (5), and the surviving eggs were kept overnight in M9-buffer on a shaker at 20 °C and the hatched L1 larvae frozen in a final concentration on 15% (w/v) glycerol in S-buffer at -80 °C.

Common garden experiment and assessment of nematode population growth rate

We performed two common garden experiment to assess the influence of the host and the microbiome on environmental adaptation. The first common garden experiment included host and microbes from six mesocosms lines (or boxes), while the second common garden experiment was focused on hosts and microbes from mesocosm boxes 1 and 2. Both common garden experiments were performed under compost conditions, while the second was additionally repeated on agar plates.

Laboratory compost consisted of approximately 10 g water-washed, grated potatoes, apples, kohlrabi, and carrots including greens placed in sterile 60 ml containers. Each laboratory compost was inoculated with 1 ml of CeMbio43 (i.e., initial microbiome) or one of the microbial communities isolated at day 100 from the mesocosms (i.e., final microbiomes), always standardized to an OD_{600}10. The containers were covered with an air-permeable film and then left for three days at room temperature, and stirred daily. The NGM agar plates were inoculated with the same CeMbio43 or final (day-100) microbial communities 24 h before the experiment. At the start of the common garden experiment, we added 100 synchronized *C. elegans* at the fourth larval stage from the initial or final host populations in M9-T to the compost or NGM plate replicates. For the second experiment, worm populations were prepared to be devoid of males, thus consisting of hermaphrodites only, in order to enhance comparability of the different *C. elegans* populations. These male-free populations were frozen in aliquots for later usage. They were rechecked for males after thawing and the subsequent initiation of worm cultures with individual L4 nematodes, followed by an assessment of the populations after 5 and 7 days, revealing and confirming the complete absence of males (Supplementary Table S1.14). Compost and plates were stored at 20 °C and, for the second common garden experiment, the compost was mixed daily by gentle shaking. After five or four days for compost or plates respectively, the worms were collected.

For the analysis of the plates, we collected worms in 5 ml M9-T, centrifuged for 1 min at 500 rpm, the supernatant was removed, and the worms washed four more times in fresh M9-T. The washed worm pellet was split in two microtubes. One microtube was stored at -20 °C until population growth rate, worm length, and worm area were determined. The other tube was stored at -80 °C.

For the compost, nematodes and microbial communities were isolated from a randomly chosen subsample following a standardized protocol. For this, the compost was mixed using a sterile wooden spatula and approximately 0.6 g compost was transferred onto a 9 cm petri dish. 15 ml sterile M9-T were

distributed over the sample by gently circling the Petri dish five times. We then collected worms in 3 x 500 µl buffer from a distance of 1 cm, 2 cm, and 3 cm around the undissolved compost sample and combined the samples into one microtube. Three technical replicates (three Petri dishes containing a 0.6 g compost subsample) were taken from each compost to compensate for variations in worm counts within a compost sample. Worm samples were frozen in 1.5 ml M9-T at -20 °C for later phenotypic analysis. Population growth rate was determined by first counting worms in 1 µl to 200 µl (depending on worm density) of the frozen sample, repeated a total of three times, and calculation of the average. Offspring per worm was calculated by dividing the count result by the 100 worms initially added to each replicate. The counted worms were extrapolated to the total frozen buffer and, for the compost experiment, to the total compost weight. Length and area of adult worms were determined for randomly selected individuals using pictures taken with a Leica stereomicroscope (Leica Microsystems GmbH, Wetzlar, Germany) and image analysis with Image J (version 2.3.0).

The two common garden experiments were performed at different time points using only minor changes in the experimental protocol (e.g., daily mixing of compost material in the second common garden experiment versus no mixing of compost material in the first common garden experiment), possibly leading to some quantitative differences in the measured traits. Importantly, for each common garden experiment, all relevant treatment groups were always assessed in parallel, under identical conditions, using a randomized distribution of samples (to minimize the influence of uncontrollable gradients in the incubators) and using neutral codes for the treatment groups (to minimize any observer bias), thereby ensuring comparability of the relevant treatments per experiment.

The population growth rates, worm length, and area of different treatment groups were compared with a Wilcoxon rank sum test and Bonferroni correction for multiple comparisons or with an ANOVA. All statistical calculations were performed with R studio software (version 2022.07.2) and can be found in Supplementary Tables S1.3, S1.5, S1.7, S1.8, S1.10, S1.12, and S1.13. Graphs were produced with R Studio and edited with Inkscape (version 1.1).

16S and ITS amplicon sequencing for microbiome analysis of common garden experiment

The compost was mixed with a sterile, wooden spatula and a compost (i.e., substrate) sample was collected and frozen at -20 °C until further use. Worms were collected from the compost sample following a previously published protocol (6). Briefly, a compost sample was covered with M9-T and emerging worms were collected using a pipette. The worms were transferred to sterile M9-T in a 3 cm Petri dish and then to a sterile microtube and kept for at least 2 min in 10 mM Tetramisole to stop ingestion and excretion of bacteria. Worms were washed another four times in fresh M9-T and frozen in 300 µl of M9-T in a 2-ml microtube in liquid nitrogen, and finally stored at -80 °C.

For isolation of bacterial DNA from nematodes, we added five to ten sterile 1 mm zirconia beads to the thawed worm samples. After crushing the samples for 3 minutes at 30 Hz, each sample was transferred to a 1.5 ml microtube and centrifuged at 8000 rpm for 3 minutes. All but 100 µl of the supernatant was removed and the pellet was resuspended in the remaining 100 µl. Using a tissue kit (Macherey-Nagel, Düren, Germany), DNA was isolated according to the manufacturer's instructions and stored at -20 °C. This protocol follows our previous methods to characterize the microbiome of *C. elegans* and we did not observe an apparent bias in the identification of the included bacteria, for example during analysis of initial inocula (7,8). Importantly, even if there was a bias, then it should have affected all treatment groups of the common garden experiment in a similar way. Therefore, the variation observed between the treatment groups of a common garden experiment should still be valid and informative.

For DNA isolation from compost, we shredded approx. 100 µl of a compost sample with 5-10 sterile 1 mm zirconia beads and 300 µl RNase-free water in a 2 ml microtube for 3 min at 30 Hz. The tubes were briefly centrifuged to spin down larger substrate particles. DNA was isolated from 100 µl compost supernatant using a modified Cetyl Trimethyl Ammonium Bromide- (CTAB) based protocol (9,10).

16S rRNA gene amplicon libraries of worm and compost DNA samples were prepared using the primers 341F (5'CCTACGGGNGGCWGCAG-3') and 806R (5'GACTACHVGGGTATCTAATCC-3') covering the V3–V4 region of the 16S rRNA gene. Libraries were sequenced on the Miseq platform using the v3-Chemie 2 × 300 bp. ITS2 libraries were prepared using the primers 5.8S-Fun (5'AACTTTYRRCAAYGGATCWCT-3') and ITS4-Fun (5'AGCCTCCGCTTATTGATATGCTTAART-3') (11). The ITS2 libraries were sequenced on the Miseq platform using the v2 nano kit 2 x 250 bp. The microbial community composition was analyzed for four replicates as well as reagent-only negative controls.

Microbiome data analysis

Raw amplicon sequencing reads were processed using the QIIME 2 v2022.2 microbiome bioinformatics platform (12). Primer and adapter sequences were removed with cutadapt (13). For the 16S amplicons, reads were filtered based on quality scores and forward and reverse reads joined with vsearch (14), and amplicon sequence variants (ASVs) were resolved with deblur (15). Taxonomy was assigned to the 16S ASVs with a naïve Bayes classifier pre-trained on the Silva 138 SSU database (16–18). For the ITS amplicons, ASVs were resolved from the forward reads with the DADA2 pipeline which removes low quality and chimeric sequences (19). Taxonomy was assigned to the ITS ASVs with a naïve Bayes classifier pre-trained on the UNITE v9.0 database (16,17,20,21). The raw reads are available from the NCBI BioProject database under the BioProject ID PRJNA954426.

All statistical analyses of the microbiome data were conducted in R Studio (22,23). Potential contaminant ASVs for both the 16S and ITS datasets were identified with the *decontam* package using the prevalence

method, which uses a statistical approach to determine the ASVs that are overrepresented in negative controls relative to experimental samples, and removed (24). We then assessed differences in microbial community composition in three main sets of analyses, which addressed the following questions:

(i) How do Box 1 and Box 2 substrate microbiomes differ in taxonomic composition, and to what degree are substrate microbiome members selected by worms? To address this question, we compared microbiome composition among substrates to which initial worms and either the initial microbiome (including the CeMbio43 bacterial community), final Box 1 microbiome, or final Box 2 microbiome were added and used differences in microbiome composition between these substrates and their respective worms as an indication of selection.

(ii) Which microbiome members are associated with the increase in fitness observed for Box 1 worms colonized by the corresponding Box 1 microbiome? To address this question, we compared microbiomes between initial worms and final Box 1 worms exposed to the final Box 1 microbiome and compared these worms to their respective substrates.

(iii) Which members of the final Box 2 microbiome are associated with low worm fitness? Here we used differences between the substrate and worm microbiomes for both final Box 2 and initial worms exposed to the final Box 2 microbiome as an indication of selection by the worm and compared the selected microbiomes of final Box 2 and initial worms exposed to the final Box 2 microbiomes.

To conduct these analyses, we first quantified dissimilarity in microbiome composition between samples with robust Aitchison distance (25) and visualized these relationships with ordinations of principal coordinates analyses. We partitioned variation in Aitchison distance among worm type, microbiome type, sample type, and their interactions and tested for significance with permutational multivariate analysis of variance (PerMANOVA) (26,27). To determine which amplicon sequence variants differed in relative abundance between pairs of treatment combinations, we used the ALDex2 R package (v 1.31.0) and considered ASVs with an effect size > 1 or < -1 to be differently abundant (28–30).

RNAseq for transcriptome analysis of *C. elegans* populations

Approximately 1000 worms were added to the compost. After 24 h, a compost subsample was placed in a 9-cm Petri dish and covered with M9-T. The emerging worms were transferred three times to fresh M9-T in a 3 cm Petri dish in as little liquid as possible to remove bacteria adhering to the worms. At least 50 worms were frozen in 800 µl TRIzol Reagent (Thermo Fisher Scientific, Waltham, MA, United States) in a 2-ml microtube in liquid nitrogen. Worms in TRIzol were thawed five times at 45 °C and refrozen in liquid nitrogen to break up the worm cuticle. Total RNA was isolated using a Direct-zol RNA MicroPrep Kit (Zymo Research, Irvine, CA, United States) following manufacturer's instructions and stored at -80 °C. The transcriptome was analyzed for five replicates. RNA libraries were prepared for sequencing using the

Illumina stranded total RNA kit with RiboZero Plus (Illumina, San Diego, USA, catalog number 20040529) according to the manufacturer's protocol. Libraries were sequenced on an Illumina NovaSeq 6000 with paired-end strategy and read length of 100 bp (Illumina, San Diego, USA, NovaSeq 6000 SP Reagent Kit v1.5 (200 cycles), catalog number 20040719). The raw data is available from the ENA database under the accession numbers ERR11455018- ERR11455037.

Transcriptome data analysis

The obtained sequence data was first processed, checked for quality, and filtered. In detail, we removed ribosomal and transfer RNA sequences and, thereafter, contaminant reads with the program BBSplit of the BBTools package v38.45 (31). Read quality trimming was performed with the BBTools package v38.45 (31), including removal of duplicate reads, adapter sequences, low-entropy reads, and trimming of bases with quality scores < 10. Reads with invalid or ambiguous bases and reads with a length < 50 base pairs (bp) were discarded. Only reads surviving quality trimming as pairs entered downstream analysis. Read quality recalibration and error correction was performed with the BBTools package v38.45 (31). The filtered reads were mapped with STAR v2.7.10b (32) to the reference genome of *C. elegans* strain N2, release WS286, retrieved from WormBase (33). We then assessed differential gene expression in three main sets of analyses, which addressed the following three main questions:

(i) To what extent do the final Box 1 and final Box 2 populations differ from each and the ancestral *C. elegans* population and thus indicate genetic evolution? To address this question, we compared variation in gene expression among the three worm populations upon exposure to the same reference microbiomes (including the CeMbio43 bacterial community) in compost, thus ensuring identical growth conditions for the three populations. Any gene expression variation observed under otherwise identical treatment conditions are most likely due to genetic differences of the worm populations.

(ii) Which gene expression changes characterize the final Box 1 worms colonized by their coexisting Box 1-day 100 microbiome and thus underlie the high population growth rate and thus the high values for a relevant fitness component expressed by this assemblage in the Box 1 common garden experiment? For this, we identified and characterized the gene expression signature that is unique to the final Box 1 worm - final Box 1 microbiome combination in comparison to the remaining three host-microbiome treatments of the Box 1 common garden experiment.

(iii) Which gene expression changes characterize the low population growth rate of worms exposed to the final Box 2 microbiome? To address this question, we compared gene expression changes in the two nematode populations exposed to the final Box 2 microbiome to those exposed to the reference microbiome.

All three sets of analyses included the following steps. At the beginning, we explored variation in gene expression across the considered treatments using a principal component analysis (PCA) with R package ggplot2 v3.4.0 (34) in R v4.2.1 (22). Thereafter, we inferred differential gene expression, always relative to the combination of the ancestral *C. elegans* population with the reference microbiome, using R package DESeq2 v1.36.0 (35). We only included genes in the analysis if they (i) have an existing Entrez gene symbol, (ii) are coding, (iii) are functional (i.e., not a pseudogene), and (iv) have ≥ 10 counts in ≥ 5 samples. Gene count distribution was modeled using a negative binomial generalized linear model. Statistical significance of the treatment effect on individual genes was determined with the Wald test (36). Effect size is expressed as log2(fold-change) of size-factor-normalized gene counts. Probabilities were adjusted using the false discovery rate (37). For the sake of clarity, only genes with a false discovery rate ≤ 0.1 are shown. Genes with absolute log2(fold-change) ≥ 1 and FDR ≤ 0.05 or 0.01, respectively, in at least one contrast, were subjected to *k*-means clustering. The optimal number of clusters was determined based on Akaike's Information Criterion. The process was repeated 100 times to account for random effects, and the median optimal number of clusters was taken as the final result. Gene clusters of contrasts with FDR ≤ 0.01 were further condensed by performing a second round of *k*-means clustering using the cluster medians of the first round as input. The C Index (38) was used to determine the optimal number of second-round clusters. We used the results of the second round of *k*-means clustering for subsequent enrichment analyses.

For enrichment analyses, we focused on individual gene clusters that showed the respective transcriptome signatures needed to address the three above questions (e.g., gene clusters indicating a difference in transcriptome response between the Box 1 and Box 2 worm populations to address question (i)). Two types of enrichment analyses were performed. On the one hand, we used the *Database for Annotation, Visualization, and Integrated Discovery* (DAVID; (39)) and studied enrichment according to Gene Ontology (GO) (40), including only categories with a probability ≤ 0.05 (after FDR correction). Cluster importance of a category was defined as Importance, following:

$$Importance_{ij} = 100 \times \frac{H_{ij}}{N_i} \times \frac{N_i}{N}$$

where H_{ij} is the number of genes in cluster i with a hit in GO category j, N_i is the total number of genes in cluster i, and N is the overall number of genes in all clusters. The rationale of this approach is to normalize the proportion of hits within each cluster by relative cluster size to avoid bias due to the latter. Cluster importance of GO categories is visualized as heatmaps.

On the other hand, we performed an enrichment analysis with the *C. elegans*-specific gene expression database WormExp (41), which contains approx. 3000 published gene expression data sets (i.e., gene sets) for *C. elegans* under diverse conditions, allowing a more taxon-specific inquiry of enriched expression

categories. Probabilities of enriched gene sets were adjusted using FDR, and the relationship of enriched gene sets was evaluated using hierarchical clustering, visualized via heatmaps. The conditions, under which the differentially expressed gene sets were obtained, yield a strong indication of their function. For example, the gene sets, which exhibit differential expression upon exposure to low doses of the toxic heavy metal cadmium, are likely involved in the stress response to cadmium. There is quite some overlap among the available transcriptome data sets, both in the considered conditions as well as the resulting gene sets, as also illustrated by the performed hierarchical clustering of enriched gene sets (Fig. S9C, S10C, S11C). Therefore, to enhance accessibility of the often complex results, we combined gene sets with such overlaps and, at the same time, with related functions or measured under related conditions, under a common header in the description of results. For example, overlapping differentially expressed gene sets obtained for different mutants, which all exhibit an increased lifespan, are summarized under the header "Lifespan". Similarly, overlapping differentially expressed gene sets, which were observed in comparisons between different natural *C. elegans* strains, are summarized under the header "Strain variation". We still provide information on the specific gene sets involved, in order to permit a fine-tuned and taxon-specific exploration of the enriched gene sets and the underlying functions, which is indeed a unique feature of the *C. elegans*-specific WormExp gene expression database (41–43).

References

1. Teotónio H, Carvalho S, Manoel D, Roque M, Chelo IM. Evolution of outcrossing in experimental populations of *Caenorhabditis elegans*. PLoS One. 2012;7:e35811.

2. Masri L, Branca A, Sheppard AE, Papkou A, Laehnemann D, Guenther PS, et al. Host–pathogen coevolution: The selective advantage of *Bacillus thuringiensis* virulence and its Cry toxin genes. Schneider DS, editor. PLoS Biol. 2015;13:e1002169.

3. Papkou A, Guzella T, Yang W, Koepper S, Pees B, Schalkowski R, et al. The genomic basis of Red Queen dynamics during rapid reciprocal host–pathogen coevolution. Proc Natl Acad Sci USA. 2019;116:923–8.

4. Carvalho S, Chelo IM, Goy C, Teotónio H. The role of hermaphrodites in the experimental evolution of increased outcrossing rates in *Caenorhabditis elegans*. BMC Evol Biol. 2014;14:116.

5. Stiernagle T. Maintenance of *C. elegans*. WormBook. 2006; Available from: http://www.wormbook.org/chapters/www_strainmaintain/strainmaintain.html

6. Petersen C, Dierking K, Johnke J, Schulenburg H. Isolation and characterization of the natural microbiota of the model nematode *Caenorhabditis elegans*. JoVE. 2022;:64249.

7. Dirksen P, Assié A, Zimmermann J, Zhang F, Tietje AM, Marsh SA, et al. CeMbio - The *Caenorhabditis elegans* microbiome resource. G3: Genes Genomes Genet. 2020;10:3025–39.

8. Zimmermann J, Piecyk A, Sieber M, Petersen C, Johnke J, Moitinho-Silva L, et al. Gut-associated functions are favored during microbiome assembly across *C. elegans* life. bioRxiv; 2023. p. 2023.03.25.534195. Available from: https://www.biorxiv.org/content/10.1101/2023.03.25.534195v1

9. Haber M. Evolutionary history of *Caenorhabditis elegans* inferred from microsatellites: Evidence for spatial and temporal genetic differentiation and the occurrence of outbreeding. Mol Biol Evol. 2005;22:160–73.

10. Schulenburg JHG, Hancock JM, Pagnamenta A, Sloggett JJ, Majerus MEN, Hurst GDD. Extreme length and length variation in the first ribosomal internal transcribed spacer of ladybird beetles (Coleoptera: Coccinellidae). Mol Biol Evol. 2001;18:648–60.

11. Taylor DL, Walters WA, Lennon NJ, Bochicchio J, Krohn A, Caporaso JG, et al. Accurate estimation of fungal diversity and abundance through improved lineage-specific primers optimized for Illumina amplicon sequencing. Cullen D, editor. Appl Environ Microbiol. 2016;82:7217–26.

12. Bolyen E, Rideout JR, Dillon MR, Bokulich NA, Abnet CC, Al-Ghalith GA, et al. Reproducible, interactive, scalable and extensible microbiome data science using QIIME 2. Nat Biotechnol. 2019;37:852–7.

13. Martin M. Cutadapt removes adapter sequences from high-throughput sequencing reads. EMBnet. 2011;17.

14. Rognes T, Flouri T, Nichols B, Quince C, Mahé F. VSEARCH: a versatile open source tool for metagenomics. PeerJ. 2016;4:e2584.

15. Amir A, McDonald D, Navas-Molina JA, Kopylova E, Morton JT, Zech Xu Z, et al. Deblur rapidly resolves single-nucleotide community sequence patterns. Gilbert JA, editor. mSystems. 2017;2:e00191-16.

16. Bokulich NA, Kaehler BD, Rideout JR, Dillon M, Bolyen E, Knight R, et al. Optimizing taxonomic classification of marker-gene amplicon sequences with QIIME 2's q2-feature-classifier plugin. Microbiome. 2018;6:90.

17. Pedregosa F, Varoquaux G, Gramfort A, Michel V, Thirion B, Grisel O, et al. Scikit-learn: Machine learning in python. MACHINE LEARNING IN PYTHON. 2012;12:2825–30.

18. Quast C, Pruesse E, Yilmaz P, Gerken J, Schweer T, Yarza P, et al. The SILVA ribosomal RNA gene database project: improved data processing and web-based tools. Nucleic Acids Research. 2012;41:D590–6.

19. Callahan BJ, McMurdie PJ, Rosen MJ, Han AW, Johnson AJA, Holmes SP. DADA2: High-resolution sample inference from Illumina amplicon data. Nat Methods. 2016;13:581–3.

20. Kõljalg U, Nilsson HR, Schigel D, Tedersoo L, Larsson KH, May TW, et al. The taxon hypothesis paradigm - on the unambiguous detection and communication of taxa. Microorganisms. 2020;8:1910.

21. Nilsson RH, Larsson KH, Taylor AFS, Bengtsson-Palme J, Jeppesen TS, Schigel D, et al. The UNITE database for molecular identification of fungi: handling dark taxa and parallel taxonomic classifications. Nucleic Acids Res. 2019;47:D259–64.

22. R Core Team. 2022. Available from: https://www.eea.europa.eu/data-and-maps/indicators/oxygen-consuming-substances-in-rivers/r-development-core-team-2006

23. R Studio Team. 2021. Available from: http://www.rstudio.com/

24. Davis NM, Proctor DM, Holmes SP, Relman DA, Callahan BJ. Simple statistical identification and removal of contaminant sequences in marker-gene and metagenomics data. Microbiome. 2018;6:226.

25. Martino C, Morton JT, Marotz CA, Thompson LR, Tripathi A, Knight R, et al. A novel sparse compositional technique reveals microbial perturbations. Neufeld JD, editor. mSystems. 2019;4:e00016-19.

26. Anderson MJ. A new method for non-parametric multivariate analysis of variance. Austral Ecology. 2001;26:32–46.

27. McArdle BH, Anderson MJ. Fitting multivariate models to community data: A comment on distance-based redundancy analysis. Ecology. 2001;82:290–7.

28. Fernandes AD, Macklaim JM, Linn TG, Reid G, Gloor GB. ANOVA-like differential expression (ALDEx) analysis for mixed population RNA-seq. PLoS ONE. 2013;8:e67019.

29. Fernandes AD, Reid JN, Macklaim JM, McMurrough TA, Edgell DR, Gloor GB. Unifying the analysis of high-throughput sequencing datasets: characterizing RNA-seq, 16S rRNA gene sequencing and selective growth experiments by compositional data analysis. Microbiome. 2014;2:15.

30. Gloor GB, Macklaim JM, Fernandes AD. Displaying variation in large datasets: Plotting a visual summary of effect sizes. J Comput Graph Stat. 2016;25:971–9.

31. Bushnell B. BBTools. 2019. Available from: http://jgi.doe.gov/data-and-tools/bbtools/

32. Dobin A, Davis CA, Schlesinger F, Drenkow J, Zaleski C, Jha S, et al. STAR: ultrafast universal RNA-seq aligner. Bioinformatics. 2013;29:15–21.

33. Davis P, Zarowiecki M, Arnaboldi V, Becerra A, Cain S, Chan J, et al. WormBase in 2022—data, processes, and tools for analyzing *Caenorhabditis elegans*. Walhout M, editor. Genetics. 2022;220:iyac003.

34. Wickham H. ggplot2: Elegant graphics for data analysis. Springer-Verlag New York; 2016. Available from: https://ggplot2.tidyverse.org/

35. Love MI, Huber W, Anders S. Moderated estimation of fold change and dispersion for RNA-seq data with DESeq2. Genome Biol. 2014;15:550.

36. Wald A. Tests of statistical hypotheses concerning several parameters when the number of observations is large. Trans Am Math Soc. 1943;54:426–82.

37. Benjamini Y, Hochberg Y. Controlling the false discovery rate: A practical and powerful approach to multiple testing. J R Stat Soc Ser B Methodol. 1995;57:289–300.

38. Hubert LJ, Levin JR. A general statistical framework for assessing categorical clustering in free recall. Psychol Bull. 1976;83:1072–80.

39. Sherman BT, Hao M, Qiu J, Jiao X, Baseler MW, Lane HC, et al. DAVID: a web server for functional enrichment analysis and functional annotation of gene lists (2021 update). Nucleic Acids Res. 2022;50:W216–21.

40. Ashburner M, Ball CA, Blake JA, Botstein D, Butler H, Cherry JM, et al. Gene Ontology: tool for the unification of biology. Nat Genet. 2000;25:25–9.

41. Yang W, Dierking K, Schulenburg H. WormExp: a web-based application for a *Caenorhabditis elegans*-specific gene expression enrichment analysis. Bioinformatics. 2016;32:943–5.

42. Yang W, Petersen C, Pees B, Zimmermann J, Waschina S, Dirksen P, et al. The inducible response of the nematode *Caenorhabditis elegans* to members of its natural microbiota across development and adult life. Front Microbiol. 2019;10.

43. Zárate-Potes A, Yang W, Pees B, Schalkowski R, Segler P, Andresen B, et al. The *C. elegans* GATA transcription factor elt-2 mediates distinct transcriptional responses and opposite infection outcomes towards different *Bacillus thuringiensis* strains. Collins JJ, editor. PLoS Pathog. 2020;16:e1008826.

Supplementary Figures

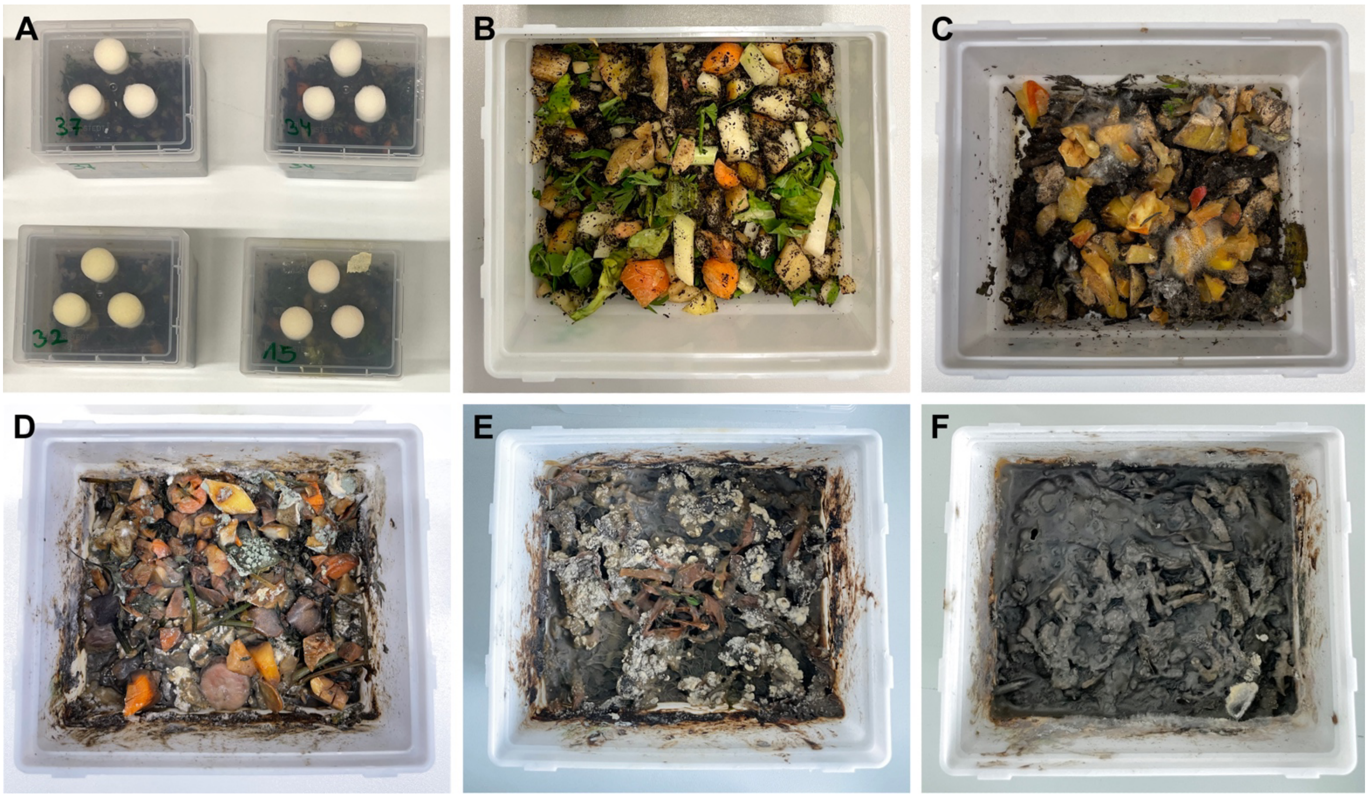

Figure S1: Mesocosm experiment in laboratory compost. (A) A mesocosm experiment was performed in boxes containing laboratory compost. **(B)** Fresh produce and compost soil were added to the boxes at the beginning of the experiment, followed by the addition of 43 native microbiota bacteria (CeMbio43) and a genetically diverse worm population. The laboratory compost was supplemented with fresh produce every two weeks. **(C)** After two weeks and **(D)** ten weeks, new microbes were visible and the compost showed signs of decomposition. On day 100, worms and microbes were harvested from **(E)** Box 1 and **(F)** Box 2.

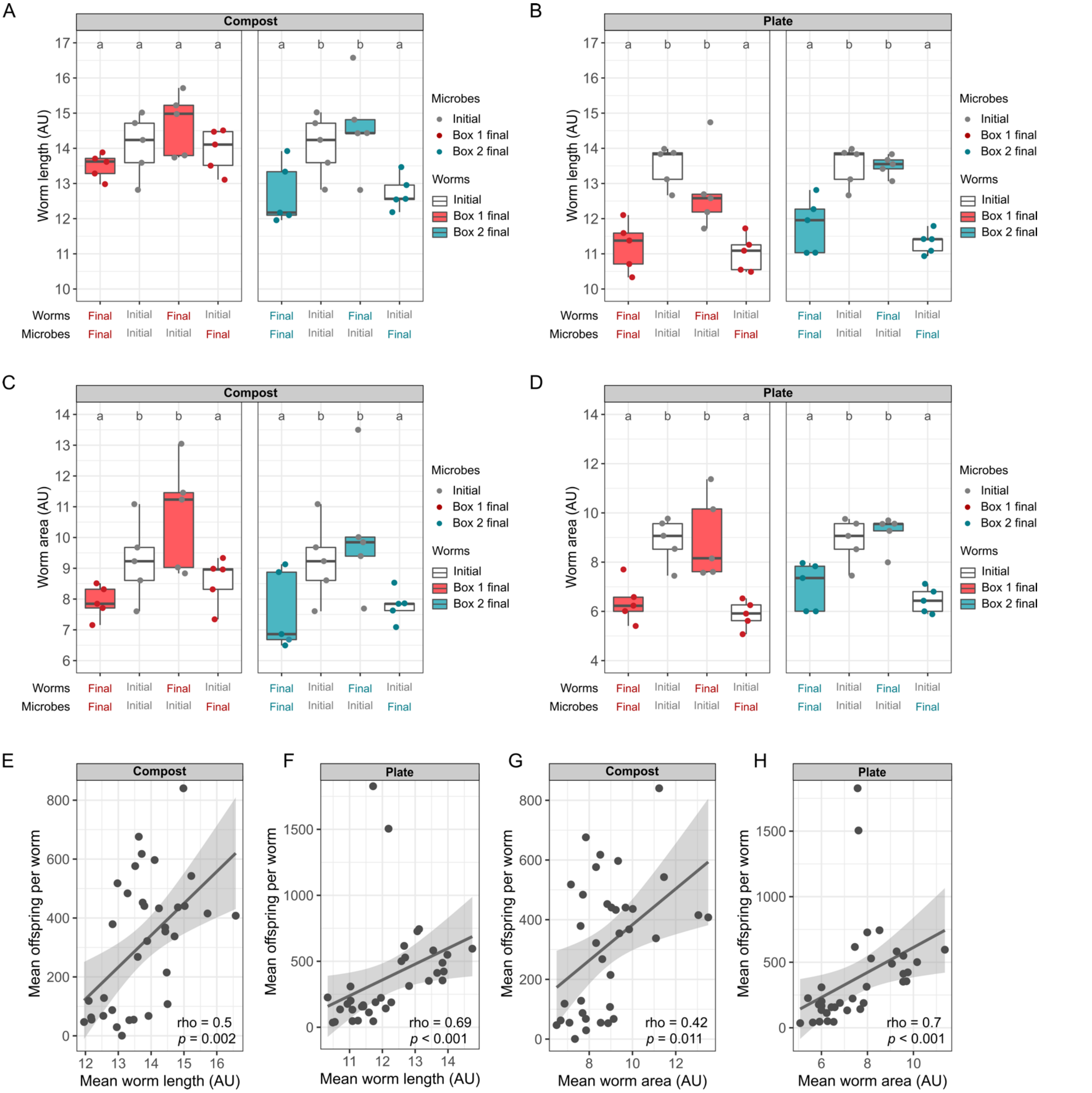

Figure S2: Host and microbiome can jointly influence proxies for nematode fitness in the novel compost environment. Results of common garden experiments, in which worm length and worm area was measured for *C. elegans* populations isolated from mesocosms at day 100 (final) and initial worms (initial) in the presence of final mesocosm microbiomes (final) or initial microbiomes including the CeMbio43 bacterial community (initial). Worm length **(A, B)** and area **(C, D)** was measured for worms from **(A, C)** compost and **(B, D)** plates. Worm length and worm area are shown in arbitrary units (AU) for final Box 1 (red boxes), final Box 2 (blue boxes), and initial worm populations (white boxes) in the presence of final Box 1 (red dots), final Box 2 (blue dots), or initial microbiomes (gray dots). Results are summarized as boxplots with the median as a thick horizontal line, the interquartile range as box, the whiskers as vertical lines, and each replicate depicted by a dot or symbol. Significant differences are indicated with different letters. n = 5. The variation in worm length and area on plates and that for Box 2 in compost are consistent with the variation in population growth rate measured under the same conditions. The variation in length and area for Box 1 in compost vary from the corresponding results for population growth rate. **(E-H)** Overall, both measures of worm body size show a significant positive correlation (assessed via Spearman rank correlation) with worm offspring numbers in compost **(E, G)** as well as on plates **(F, H)**, supporting the suggestion that both are related and that worm size is a meaningful proxy for worm fitness. Lines are predicted from a linear model. Shaded areas indicate the 95% confidence interval.

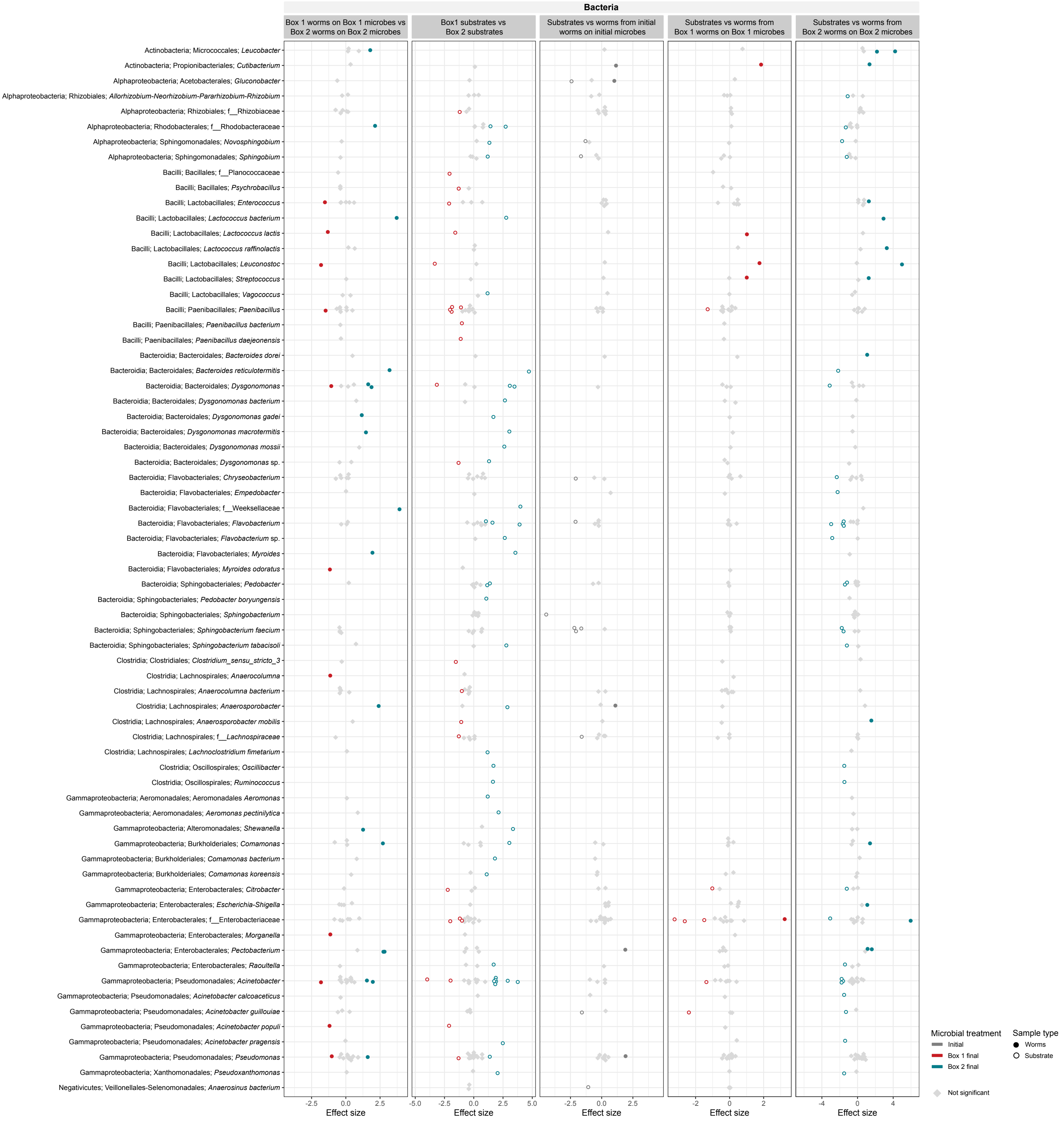

Figure S3: Microbiome treatments resulted in differences in both compost and nematode microbiomes. Pairwise differential abundance analyses of worm/substrate bacterial microbiomes (final Box 1, final Box 2 or initial inoculum including the CeMbio43 bacterial community). Each point represents an ASV and ASVs are grouped by genus with class and order listed. Colors and shapes indicate treatment and sample type of differentially abundant ASVs.

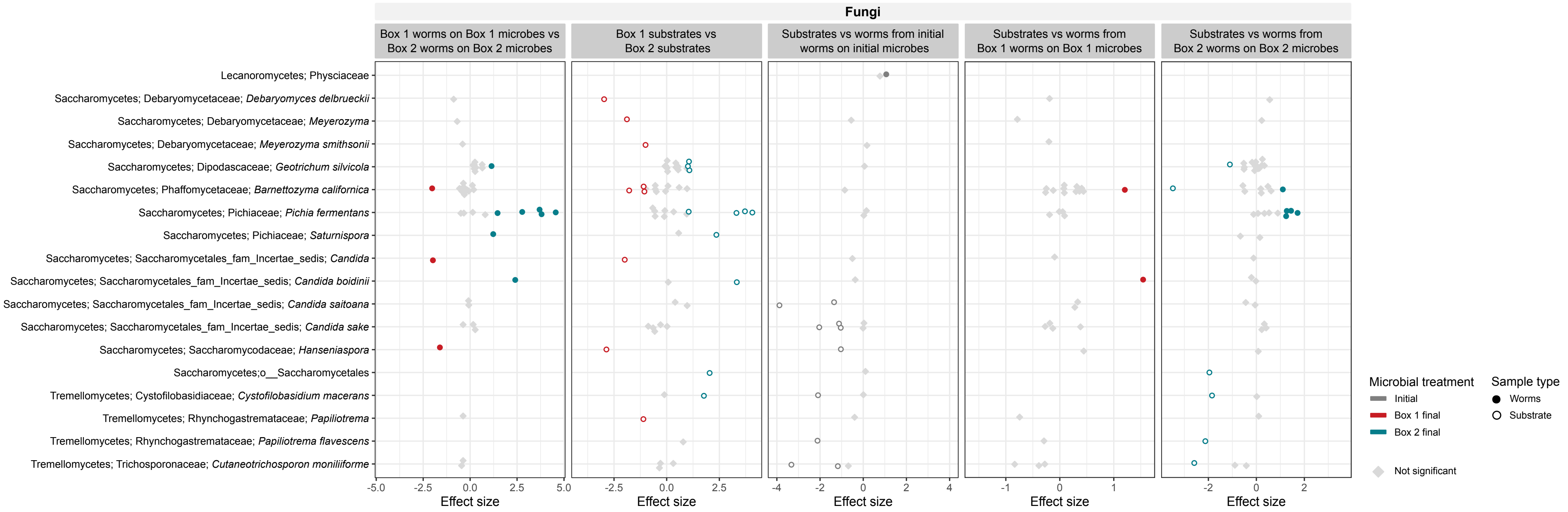

Figure S4: Microbiome treatments resulted in differences in both compost and nematode microbiomes. Pairwise differential abundance analyses of worm/substrate fungal microbiomes (final Box 1, final Box 2 or initial inoculum including the CeMbio43 bacterial community). Each point represents an ASV and ASVs are grouped by genus with class and family listed. Colors and shapes indicate treatment and sample type of differentially abundant ASVs.

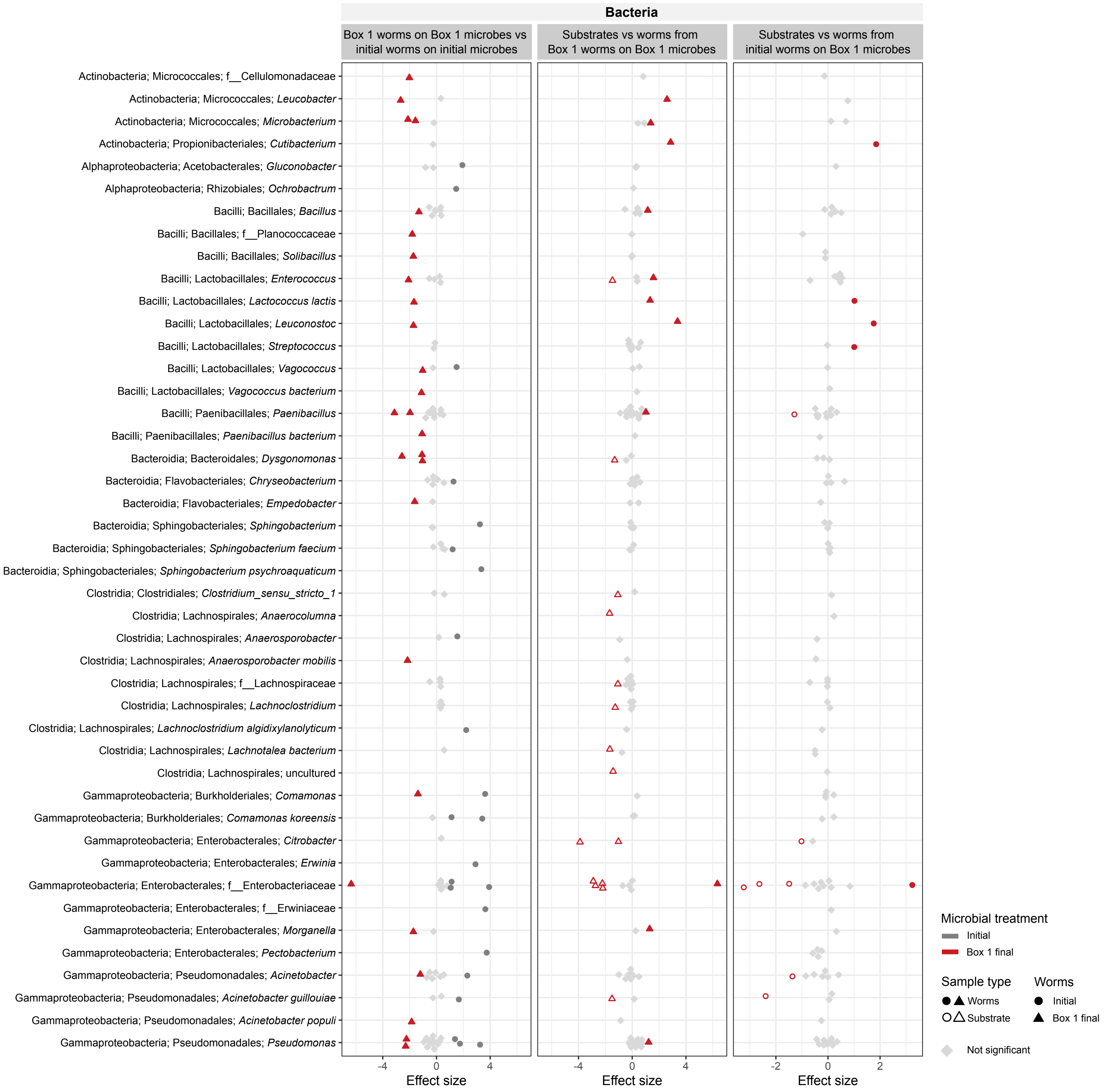

Figure S5: Differences in microbiome composition were associated with increased fitness in nematodes from the Box 1 mesocosm. Pairwise differential abundance analyses of bacterial microbiomes from initial or final Box 1 worms/substrates exposed to final Box 1 inoculum or initial inoculum including the CeMbio43 bacterial community. Each point represents an ASV and ASVs are grouped by genus with class and order listed. Colors and shapes indicate treatment and sample type of differentially abundant ASVs.

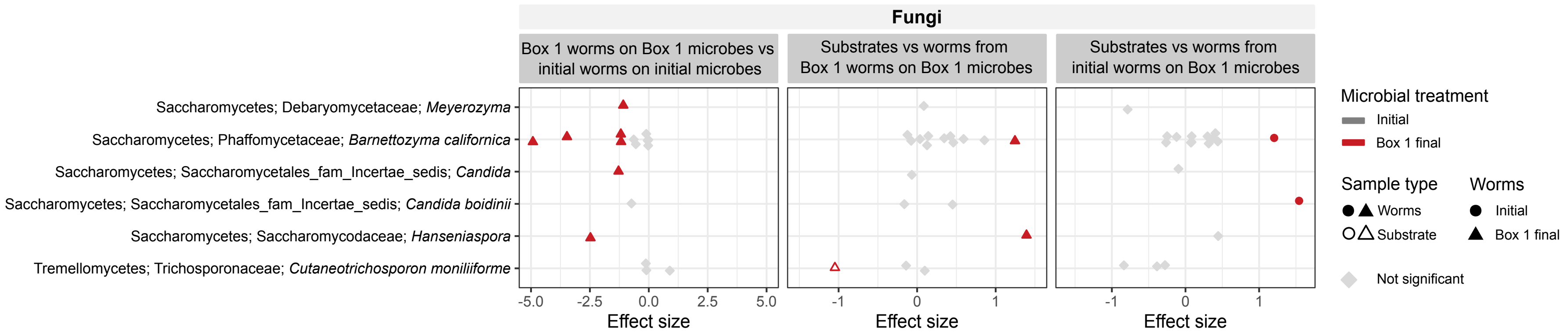

Figure S6: Differences in microbiome composition were associated with increased fitness in nematodes from the Box 1 mesocosm. Pairwise differential abundance analyses of fungal microbiomes from initial or final Box 1 worms/substrates exposed to final Box 1 inoculum or initial inoculum including the CeMbio43 bacterial community. Each point represents an ASV and ASVs are grouped by genus with class and family listed. Colors and shapes indicate treatment and sample type of differentially abundant ASVs.

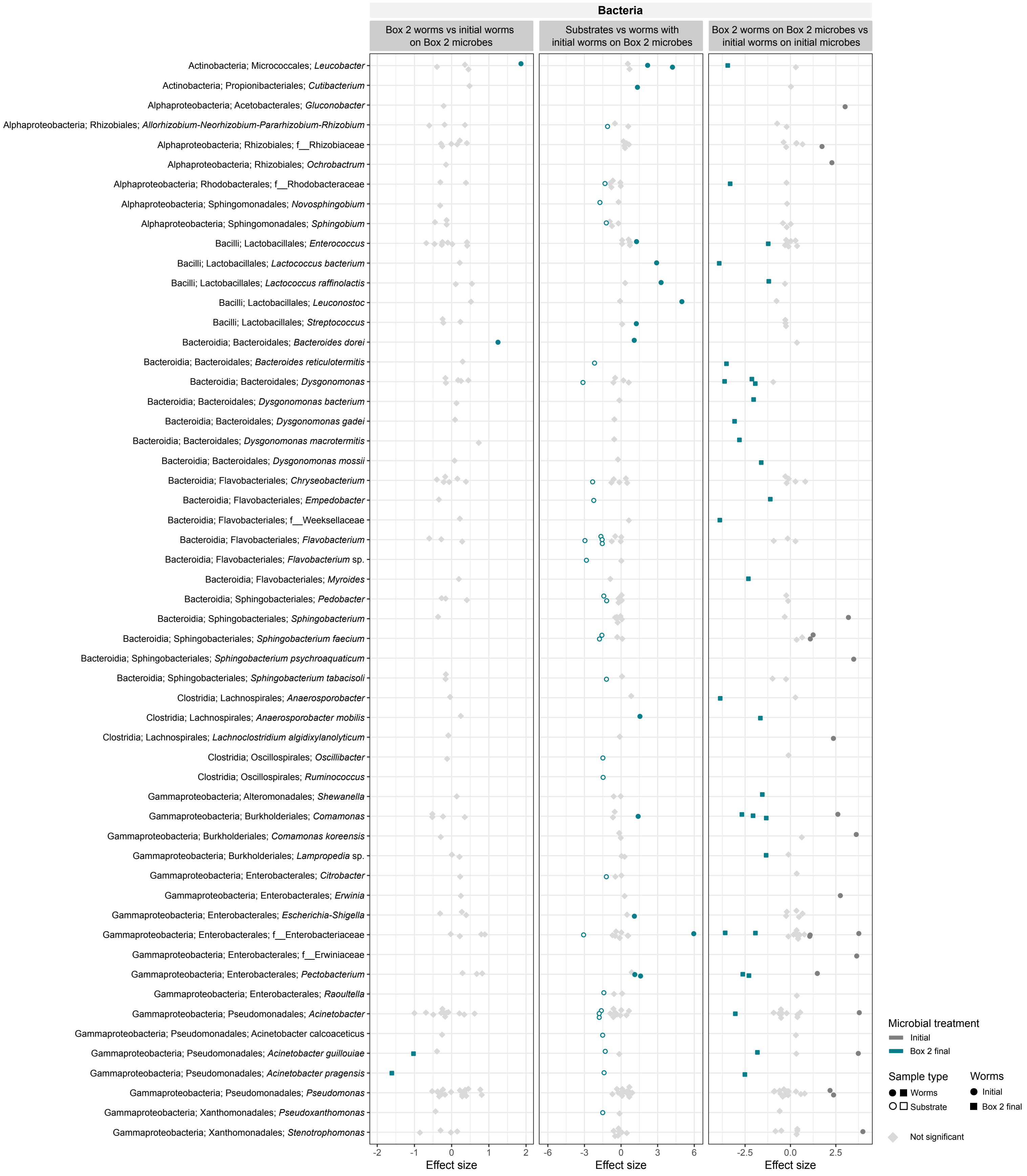

Figure S7: Differences in microbiome composition were associated with decreased fitness in nematodes from the Box 2 mesocosm. Pairwise differential abundance analyses of bacterial microbiomes from initial or final Box 2 worms/substrates exposed to final Box 2 inoculum or initial inoculum including the CeMbio43 bacterial community. Each point represents an ASV and ASVs are grouped by genus with class and order listed. Colors and shapes indicate treatment and sample type of differentially abundant ASVs.

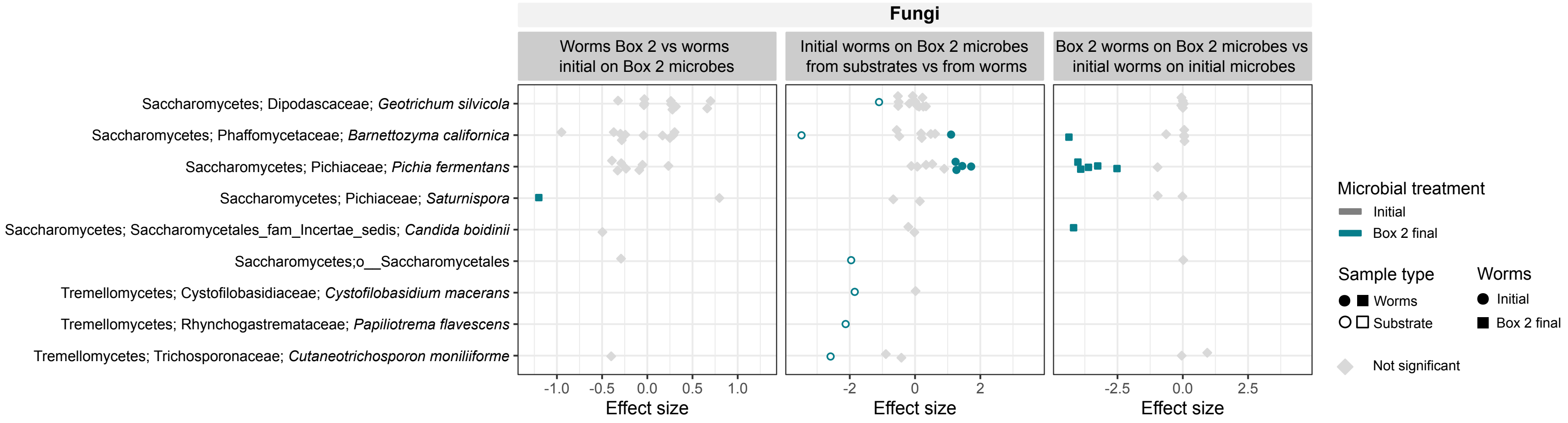

Figure S8: Differences in microbiome composition were associated with decreased fitness in nematodes from the Box 2 mesocosm. Pairwise differential abundance analyses of fungal microbiomes from initial or final Box 2 worms/substrates exposed to final Box 2 inoculum or initial inoculum. Each point represents an ASV and ASVs are grouped by genus with class and family listed. Colors and shapes indicate treatment and sample type of differentially abundant ASVs.

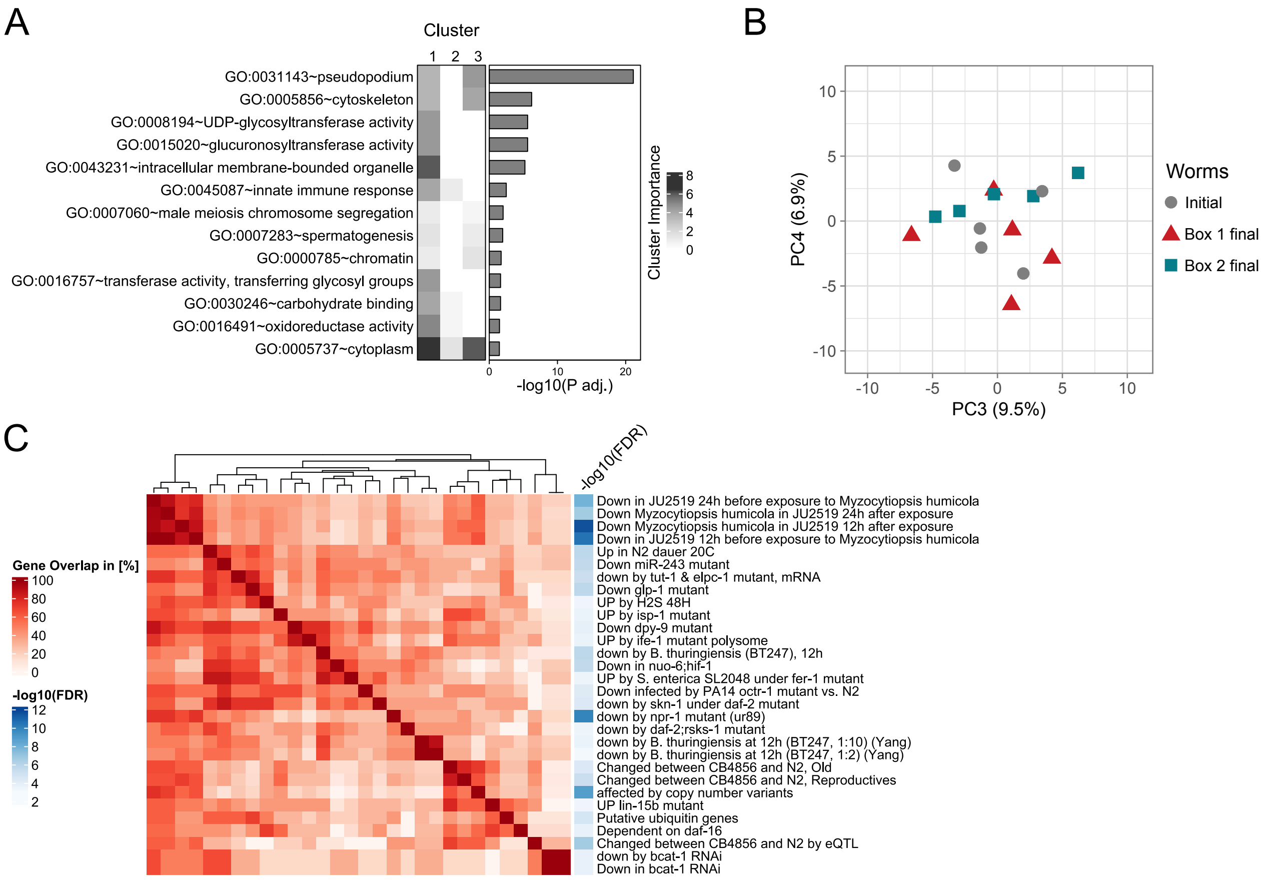

Figure S9: Differential gene expression in the adapted Box 1 *C. elegans* populations. Transcriptome data analysis for the comparison of initial, final Box 1, and final Box 2 *C. elegans* populations assayed under identical compost conditions with initial microbiomes including the CeMbio43 bacterial community. **(A)** Enriched gene ontology (GO) terms of differentially expressed genes. GO enrichment analysis was performed by DAVID. **(B)** General variation in gene expression was explored with a principal component analysis, whereby the panel shows the spread of sample variation along the third and fourth principal components (PC3, PC4). Symbols and colors indicate final Box 1 (red triangle), final Box 2 (blue square), or initial (gray circle) worm origin. n = 5. **(C)** shows the results of the focused enrichment analysis of cluster 2 with the *C. elegans*-tailored WormExp database and visualization of differential expression using heatmaps, whereby the heatmaps always show the gene overlap in percent. Description on the right gives terms of the gene functions and fold change after FDR correction.

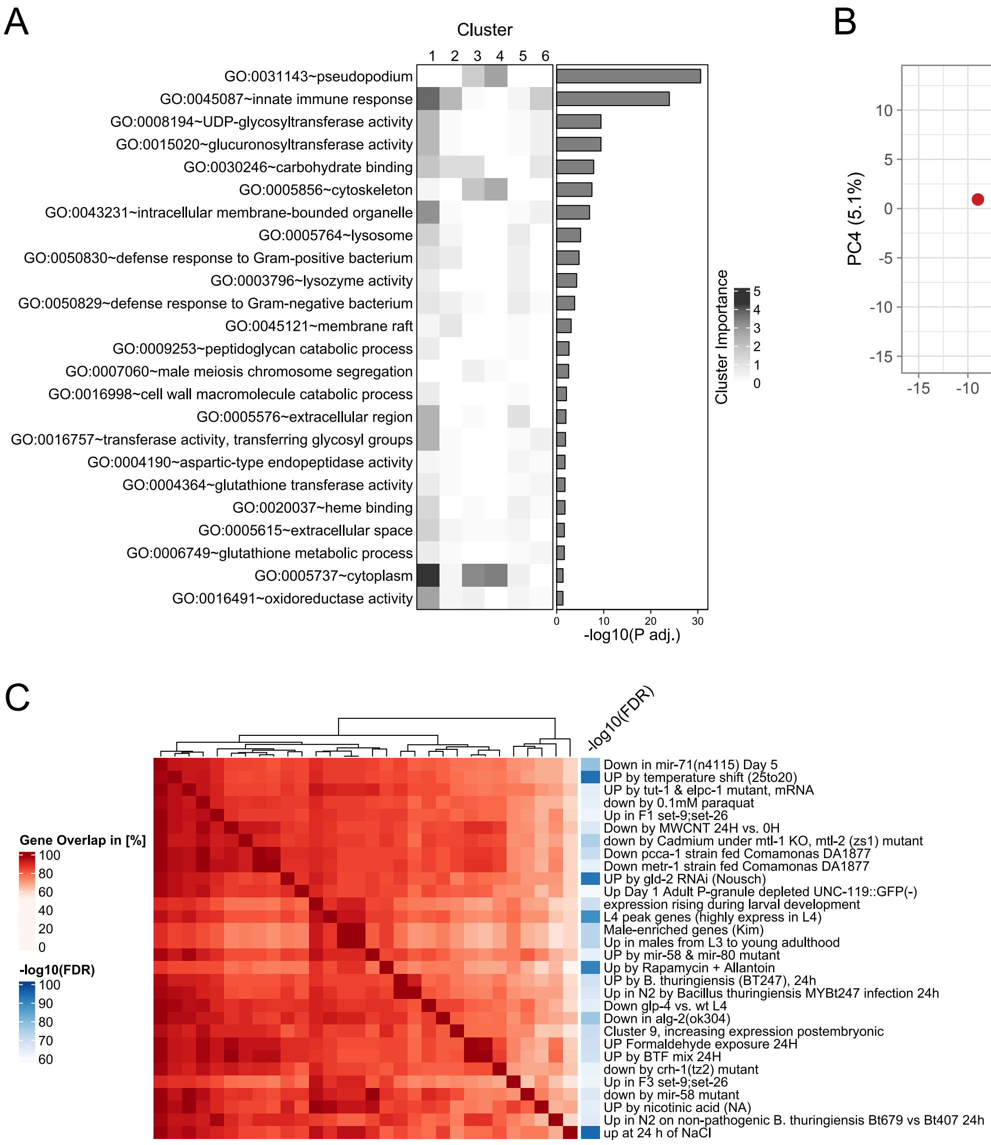

Figure S10: Differential gene expression in the adapted Box 1 *C. elegans* populations. Transcriptome data analysis for the comparison of all possible host-microbiome combinations for the Box 1 common garden experiment. Initial or final Box 1 worms were combined with either the initial microbes (including the CeMbio43 bacterial community) or the final Box 1 microbes. **(A)** Enriched gene ontology (GO) terms of differentially expressed genes. GO enrichment analysis was performed by DAVID. **(B)** General variation in gene expression was explored with a principal component analysis, whereby the panel shows the spread of sample variation along the third and fourth principal components (PC3, PC4). Symbols indicate final Box 1 (triangle) or initial (circle) worm origin; colors indicate final Box 1 (red) or initial (gray) microbiome origin. n = 5. **(C)** shows the results of the focused enrichment analysis of cluster 3 with the *C. elegans*-tailored WormExp database and visualization of differential expression using heatmaps, whereby the heatmaps always show the gene overlap in percent. Description on the right gives terms of the gene functions and fold change after FDR correction.

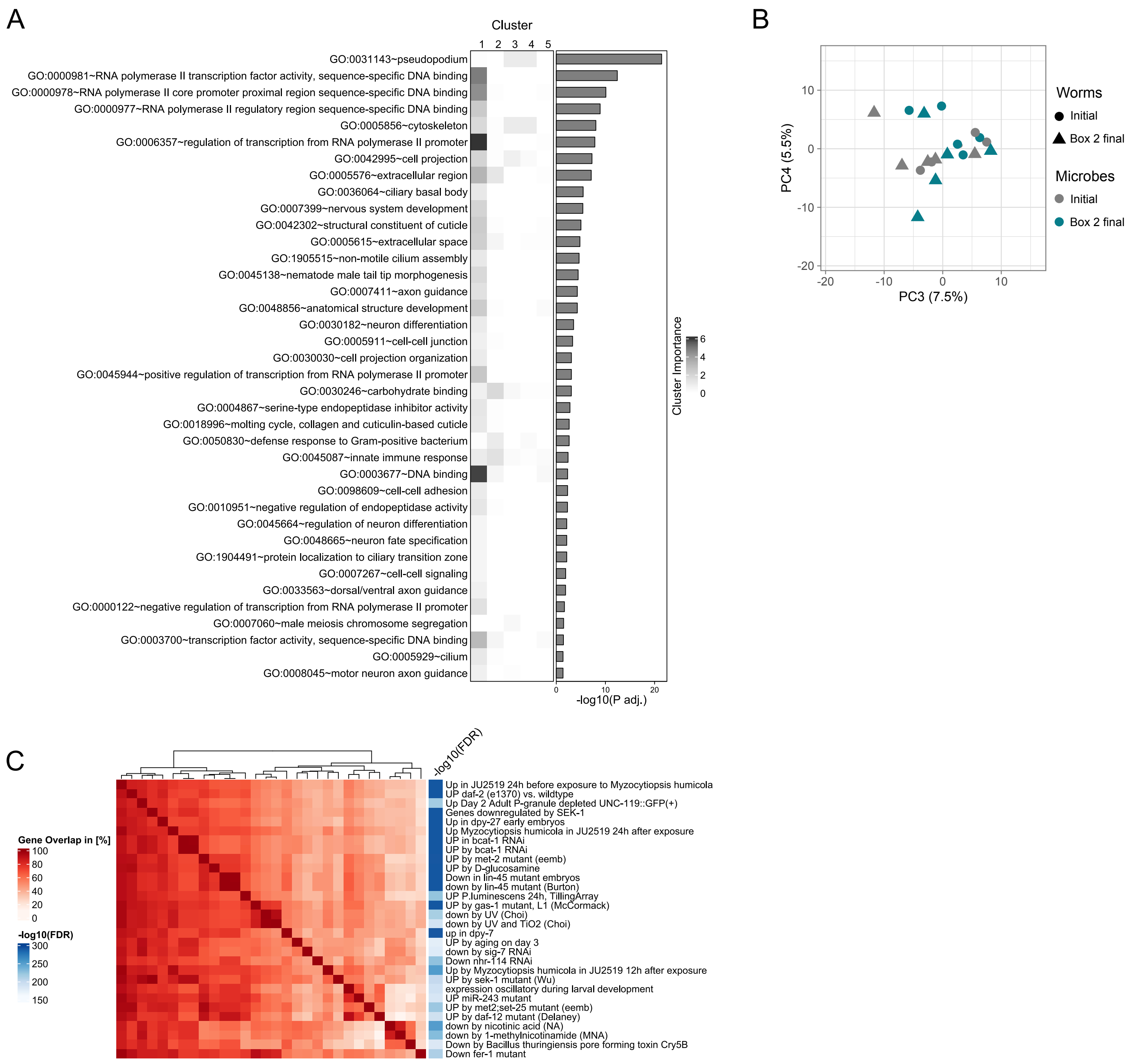

Figure S11: Differential gene expression in the adapted Box 2 *C. elegans* populations. Transcriptome data analysis for the comparison of all possible host-microbiome combinations for the Box 2 common garden experiment. Initial or final Box 2 worms were combined with either the initial microbes (including the CeMbio43 bacterial community) or the final Box 2 microbes. **(A)** Enriched gene ontology (GO) terms of differentially expressed genes. GO enrichment analysis was performed by DAVID. **(B)** General variation in gene expression was explored with a principal component analysis, whereby the panel shows the spread of sample variation along the third and fourth principal components (PC3, PC4). Symbols indicate final Box 2 (triangle) or initial (circle) worm origin; colors indicate final Box 2 (blue) or initial (gray) microbiome origin. n = 5. **(C)** shows the results of the focused enrichment analysis of cluster 1 with the *C. elegans*-tailored WormExp database and visualization of differential expression using heatmaps, whereby the heatmaps always show the gene overlap in percent. Description on the right gives terms of the gene functions and fold change after FDR correction.
