# Supplementary material for: Host and microbiome jointly contribute to environmental adaptation
Source: ISME J. 2023 Sep 6;17(11):1953–65. doi: 10.1038/s41396-023-01507-9 (PMC10579302; doi:10.1038/s41396-023-01507-9)
Supplement: Supplementary file 2 — Supplementary Information 2 [file 41396_2023_1507_MOESM2_ESM.pdf]

Supplementary Information 2

Host and microbiome jointly contribute to environmental adaptation

Carola Petersen[1*], Inga K. Hamerich[1*], Karen L. Adair[2*], Hanne Griem-Krey[1], Montserrat Torres Oliva[3], Marc P. Hoeppner[3], Brendan J.M. Bohannan[2†], Hinrich Schulenburg[1,4 †]

[*]Shared first authors

[†]Shared senior authors

Corresponding author:

h.schulenburg@zoologie.uni-kiel.de

[1] Department of Evolutionary Ecology and Genetics, Kiel University, Kiel, Germany

[2] Institute of Ecology and Evolution, University of Oregon, Eugene, OR, USA

[3] Institute of Clinical Molecular Biology, Kiel University, Kiel, Germany

[4] Max-Planck Institute for Evolutionary Biology, Ploen, Germany

Supplementary Files

Supplementary Information 1 (PDF)

Material and methods including details of nematode and bacterial strains, mesocosm experiment, common garden experiment, assessment of population growth rate, 16S rRNA gene, and ITS amplicon sequencing, microbiome data analysis, RNAseq for transcriptome analysis, and transcriptome data analysis. Supplementary Figures S1-S11 showing results for worm length/area, microbiome composition, and differential gene expression.

Supplementary Table S1 (MS Excel)

Supplementary Tables S1.1 to S1.14 with information regarding species included in the CeMbio43 bacterial community, data for population growth rates, worm length, and worm area from common garden experiments, male frequencies, test statistics for population growth rates, worm length, and worm area from common garden experiments.

Supplementary Table S2 (MS Excel)

Supplementary Tables S2.1 to S2.5 including read counts of fungal (ITS2) and bacterial (16S rRNA gene) ASVs from common garden experiment, and results of PerMANOVA tests for differences in microbiomes.

Supplementary Table S3 (MS Excel)

Supplementary Tables S3.1 to S3.6 including information on condensed clusters of differentially expressed genes and results of the enrichment analysis with the *C. elegans*-tailored WormExp database.

Supplementary Movie 1 (QuickTime Movie)

Video of a sample from the upper compost layer at day 244 of the mesocosm experiment showing large numbers of proliferating worms.

Supplementary Movie 2 (QuickTime Movie)

Video of worms in from mesocosm experiment in M9-buffer. The worms are released from a compost sample after the addition of M9-buffer and can thus be secured for further analysis.

Supplementary Movie 3 (QuickTime Movie)

Video of worms from mesocosm experiment in M9-buffer. The worms are released from a compost sample after the addition of M9-buffer and can thus be secured for further analysis.
